# A Cdh1–FoxM1–Apc axis controls muscle development and regeneration

**DOI:** 10.1038/s41419-020-2375-6

**Published:** 2020-03-09

**Authors:** Zhe Chen, Lei Li, Shuangnian Xu, Zhilong Liu, Chengfang Zhou, Zhigang Li, Yuanyuan Liu, Weiru Wu, Yongxiu Huang, Mei Kuang, Shijun Fan, Hui Li, Xi Li, Guanbin Song, Wen-Shu Wu, Jieping Chen, Yu Hou

**Affiliations:** 1 Department of Hematology, Southwest Hospital, Third Military Medical University (Army Medical University), 400038 Chongqing, China; 2 Medical Research Center, Southwest Hospital, Third Military Medical University (Army Medical University), 400038 Chongqing, China; 3 Key Laboratory of Biorheological Science and Technology, Ministry of Education, College of Bioengineering, Chongqing University, 400044 Chongqing, China; 4 Department of Medicine, College of Medicine, University of Illinois at Chicago, Chicago, IL 60612 USA

**Keywords:** Cell proliferation, Muscle stem cells

## Abstract

Forkhead box M1 (FoxM1) transcriptional factor has a principal role in regulating cell proliferation, self-renewal, and tumorigenesis. However, whether FoxM1 regulates endogenous muscle development and regeneration remains unclear. Here we found that loss of FoxM1 in muscle satellite cells (SCs) resulted in muscle atrophy and defective muscle regeneration. FoxM1 functioned as a direct transcription activator of adenomatous polyposis coli (Apc), preventing hyperactivation of wnt/β-catenin signaling during muscle regeneration. FoxM1 overexpression in SCs promoted myogenesis but impaired muscle regeneration as a result of spontaneous activation and exhaustion of SCs by transcriptional regulation of Cyclin B1 (Ccnb1). The E3 ubiquitin ligase Cdh1 (also termed Fzr1) was required for FoxM1 ubiquitylation and subsequent degradation. Loss of Cdh1 promoted quiescent SCs to enter into the cell cycle and the SC pool was depleted by serial muscle injuries. Haploinsufficiency of FoxM1 ameliorated muscle regeneration of Cdh1 knock-out mice. These data demonstrate that the Cdh1–FoxM1–Apc axis functions as a key regulator of muscle development and regeneration.

## Introduction

Skeletal muscle is a homeostatic tissue that requires myogenesis upon postnatal growth and muscle regeneration following an injury^1^. Myogenesis is a tightly controlled process including muscle stem cells (also called muscle satellite cells (SCs)), which proliferate, differentiate to myoblasts, and subsequently form multinucleated myotubes^2,3^. A portion of activated SCs return to quiescence and replenish the SC pool^4^. The balanced transition of SCs between quiescence, proliferation, and differentiation is critical for SC maintenance and muscle homeostasis.

Muscle-specific transcriptional factors, including Pax3, Pax7, Mfy5, MyoD, and Myogenin, play critical roles in the determination of the fate of SCs, as well as in muscle development and regeneration^5^. Several key components of SC niche, such as laminin-α1 and collagen VI, are necessary for self-renewal of SCs and muscle regeneration^6,7^. Notch signaling, as well as p38/mitogen-activated protein kinase, wnt, phosphatase and tensin homolog, and several cell cycle regulators have been shown to regulate SC expansion and muscle development^8–11^. Recently, the ubiquitin–proteasome system has been demonstrated to be essential for the maintenance of SCs. Knockout of the Rp13 proteasomal component impairs the ability of SCs to proliferate, survive, and differentiate, resulting in defective muscle regeneration^12^. The anaphase-promoting complex/cyclosome (APC/C) is a multisubunit E3 ubiquitin ligase complex that regulates cyclic degradation of cell cycle regulator via the Cdh1 or Cdc20 adaptor proteins^13^. APC/C^Cdh1^ has a broad spectrum of substrates in and beyond the cell cycle^14,15^. However, the role of APC/C^Cdh1^ in SC maintenance is unclear.

Forkhead box M1 (FoxM1) is one of the substrates of APC/C^Cdh1^ in several cancer cells^16,17^ and is a well-established regulatory transcriptional factor for cell cycle progression^18^. FoxM1 has been reported to play critical roles in stem cells^19,20^. FoxM1 is essential for proliferation of human embryonic stem cells and protects them against oxidative stress by transcriptionally regulating Cyclin B1 (Ccnb1) and cyclin-dependent kinase 1 (Cdk1)^19^. The higher expression of FoxM1 is essential for the maintenance of hematopoietic stem cells by transcriptionally regulating Nurr1^20^. We previously described that FoxM1 promotes the proliferation and survival of C2C12 cells (an immortalized myoblast cell line) in vitro partially by the involvement of long noncoding RNAs^21^. FoxM1 expression displays dynamic changes during the transition of C2C12 between quiescence and activation^21^, implying the important roles of FoxM1 in SCs. However, the precise functions of FoxM1 in SCs and whether FoxM1 protein is a substrate of APC/C^Cdh1^ are largely unknown.

Besides its transcriptional regulation of target genes, FoxM1 has been reported to directly interact with proteins, such as Npm1^22^, Rnf168^23^, Melk^24^, and beta (β)-catenin^25,26^. β-Catenin is the core factor of the wnt pathway. Degradation of β-catenin is promoted by a cytoplasmic destruction complex consisting of adenomatous polyposis coli (Apc), Axin, Cki, and Gsk3β, which phosphorylates β-catenin^27^. Conversely, FoxM1 binds directly to β-catenin and enhances β-catenin nuclear localization in glioma cell lines^25^ and pancreatic cancer cells^26^. The constitutive activation of β-catenin in mice has no obvious effect on the amount of SCs under homeostatic conditions, but these mice show muscle regeneration defects with aberrant myofiber morphology and atrophy, which is mainly attributed to hyperactive transforming growth factor-beta (TGF-β) signaling^28^. However, little is about the role of FoxM1 in muscle regeneration and the correlation between FoxM1 and wnt signaling in SCs.

In this study, we demonstrate that the loss of FoxM1 results in muscle atrophy and damaged muscle regeneration. Both FoxM1 deficiency and overexpression impaired SC maintenance. Unexpectedly, FoxM1 promoted β-catenin degradation by transcriptionally regulating Apc. Moreover, the accumulation of FoxM1 protein was controlled by Cdh1 via ubiquitylation. The identified Cdh1–FoxM1–Apc regulatory axis provides new insight on the regulators of SC maintenance and muscle homeostasis.

## Results

### FoxM1 deficiency results in muscle atrophy and impairs muscle regeneration

**Fig. 1:**
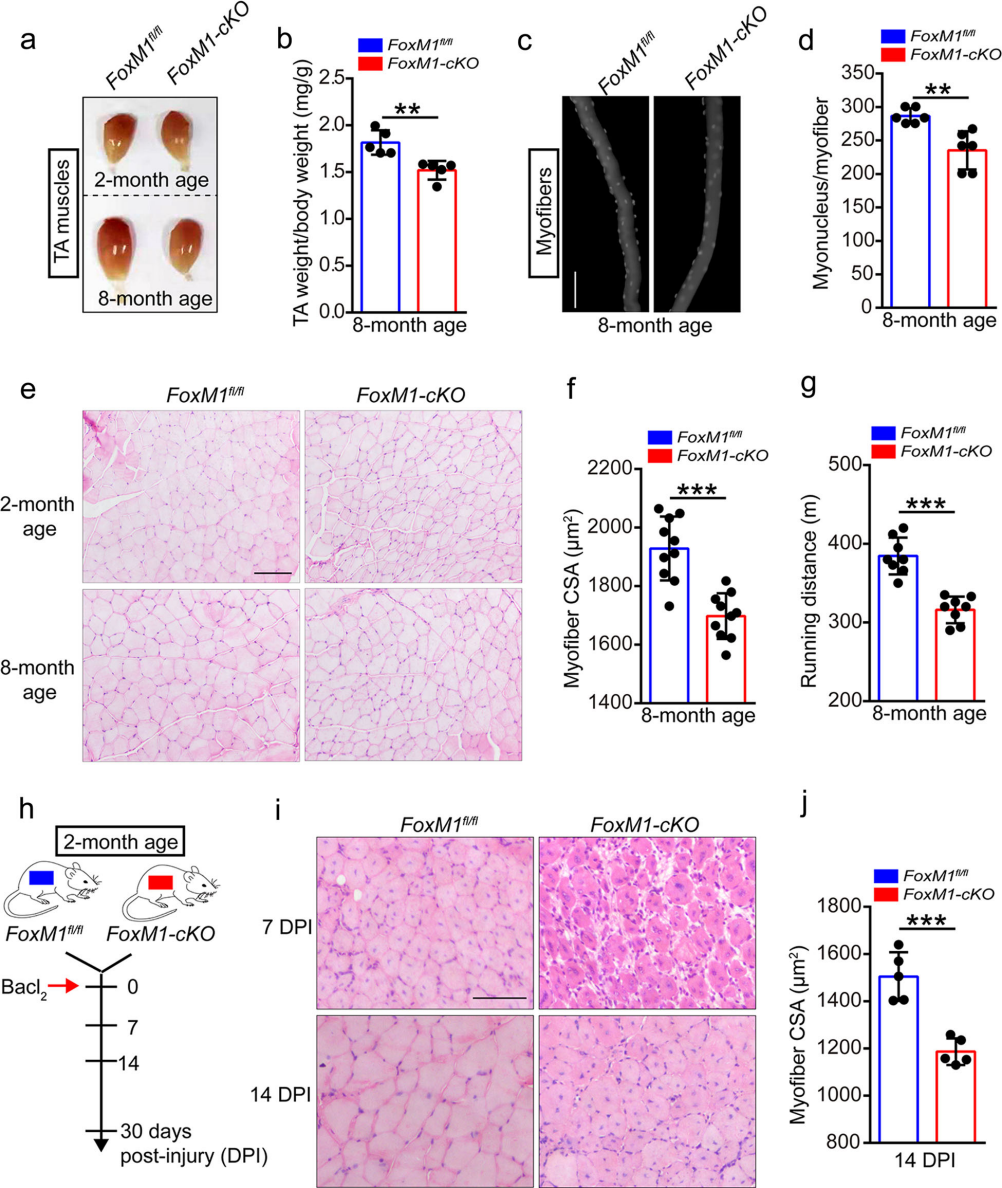
FoxM1 deficiency results in muscle atrophy and impairs muscle regeneration. **a** The visual comparison of muscle mass of tibialis anterior (TA) in FoxM1 deletion mice compared with control mice at 2 or 8 months of age. **b** Quantification of TA weight/body weight in *FoxM1*^*fl/fl*^ and *FoxM1-cKO* mice at 8 months of age (*n* = 5). **c**, **d** Immunofluorescence of myonucleus on single myofiber by DAPI staining (**c**) and number count (**d**) (*n* = 6, 20 myofibers per mouse). Scale bar, 100 μm. **e** Hematoxylin and eosin (HE) staining of the TA muscle cross-sections in mice at 2 or 8 months of age. Scale bar, 100 μm. **f** Average cross-section area (CSA) of TA in *FoxM1*^*fl/fl*^ and *FoxM1-cKO* mice at 8 months of age (*n* = 10). **g** The maximum running distance (measured by a treadmill) of *FoxM1-cKO* mice compared with control mice at 8 months of age (*n* = 8). **h** A schematic illustration showing the experimental design for muscle injury. BaCl_2_ was injected into and along the length of TA and gastrocnemius muscle of mice (2 months of age). The skeletal muscle or TA muscle was harvested at 7–30 days post-injury (DPI) depending on the experiments. **i** HE staining of the TA muscle cross-sections of *FoxM1-cKO* mice compared with control mice at 7 days and 14 DPI. Scale bar, 100 μm. **j** Average CSA of TA muscle in mice at 14 DPI (*n* = 5). Error bars represent the means ± SD. **p < 0.01, ***p < 0.001; Student’s *t* test.

To determine whether FoxM1 regulates muscle homeostasis, we crossed mice with loxP-flanked FoxM1 alleles (*FoxM1*^*fl/fl*^)^29^ with *Pax7-Cre* mice^30^ to generate control *FoxM1*^*fl/fl*^ mice and *FoxM1*^*fl/fl*^*Pax7-Cre* mice (designated here as “*FoxM1-cKO* mice”) (Supplementary Fig. S1a, b). The expression of FoxM1 in SCs was efficiently knocked out (Supplementary Fig. 1c, d). The weight of tibialis anterior (TA) muscle in *FoxM1-cKO* mice showed no obvious differences with *FoxM1*^*fl/fl*^ mice at 2 months of age but showed decreased weight at 8 months of age (Fig. 1a, b and Supplementary Fig. 1e). Moreover, the myofibers of extensor digitorum longus (EDL) in *FoxM1*^*fl/fl*^*-cKO* mice showed reduced numbers of myonuclei/myofiber than *FoxM1*^*fl/fl*^ mice at 8 months of age (Fig. 1c, d and Supplementary Fig. 1f). Histological analysis of TA revealed muscle atrophy with age in *FoxM1-cKO* mice compared with control mice (Fig. 1e, f and Supplementary Fig. 1g). *FoxM1-cKO* mice were inferior in the maximum running distance compared with control mice at 8 months of age (Fig. 1g and Supplementary Fig. 1h). These data suggested that loss of FoxM1 in SCs resulted in muscle loss with age.

To explore the effect of FoxM1 deficiency on muscle regeneration, we induced muscle injury by injecting BaCl_2_ into muscles of mice at 2 months of age (Fig. 1h) Histological analysis of the TA muscles at 7 days and 14 days after injury revealed a more severe regeneration defect in *FoxM1*-*cKO* mice, as evidenced by the larger unrepaired areas (Fig. 1i). Deletion of FoxM1 in SCs resulted in smaller-sized regenerative myofibers, compared with control mice at 14 days post-injury (DPI) (Fig. 1j). Together, these data suggested that loss of FoxM1 in SCs resulted in muscle atrophy and impaired muscle regeneration.

### FoxM1 deficiency impairs SC maintenance by impeding cell cycling of SCs

**Fig. 2:**
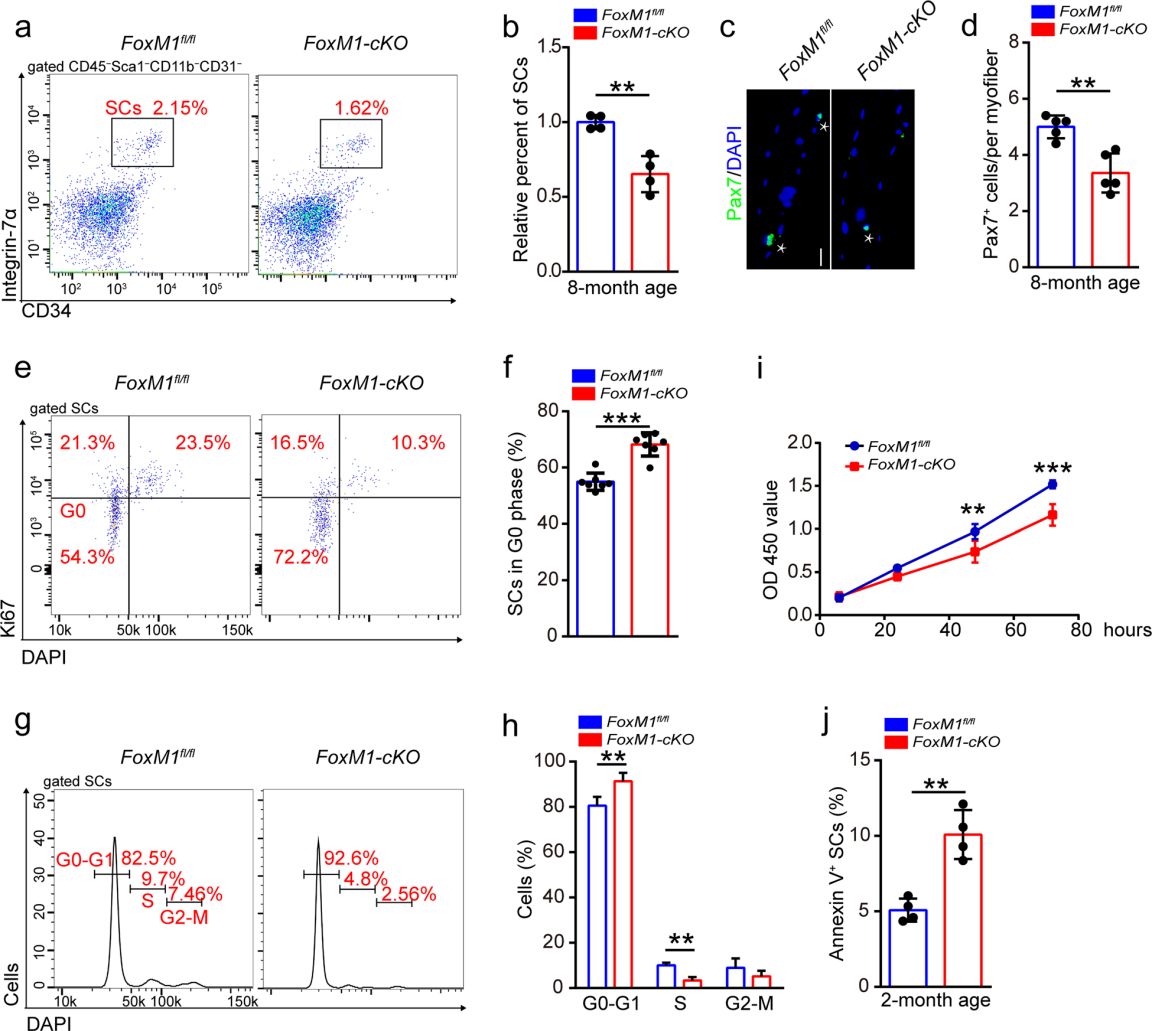
FoxM1 deficiency impairs SC maintenance by impeding cell cycling of SCs. **a** Skeletal muscles were harvested from mice at 8 months of age and then were digested as mononuclear cells. The cells were stained with cell surface markers (CD45, Sca1, CD11b, CD31,CD34, α7-integrin). The population of SCs (defined as CD45^−^Sca1^−^CD11b^−^CD31^−^CD34^+^α7-integrin^+^ cells) was analyzed by flow cytometry. **b** Quantification of the relative percentage of SCs per total mononuclear cells isolated from the skeletal muscles of mice at 8 months of age (*n* = 4). **c** Skeletal muscles were harvested from mice (8 months of age) and digested as myofibers. Representative fluorescent images of fresh myofibers immunostained with Pax7 antibody (green) and DAPI (blue). Scale bars, 50 μm. **d** Quantification of the numbers of Pax7^+^ SCs per myofiber of mice at 8 months of age (*n* = 5, 20 myofibers per mouse). **e** The mononuclear cells were isolated from the skeletal muscles of mice (2 months of age) and stained with cell surface markers, DAPI and Ki67. The quiescent percentage of SCs was analyzed by flow cytometry. **f** Quantification of the percentage of G0 phase of SCs in mice (2 months of age) (*n* = 7). **g** The mononuclear cells were isolated from the skeletal muscles of mice (2 months of age) and were stained with cell surface markers and DAPI. The cell cycle of SCs was analyzed by flow cytometry. **h** Quantification of the percentage of cell cycle distribution of SCs in mice at 2 months of age (*n* = 4). **i** In vitro proliferation assay (CCK8) of cultured SCs isolated from mice (2 months of age; *n* = 5). **j** The mononuclear cells were isolated from the skeletal muscles of mice (2 months of age) and were stained with SC markers, as well as Annexin-V and DAPI. The percentage of apoptotic SCs (Annexin-V^+^ SCs) was analyzed by flow cytometry (*n* = 4). Error bars represent the means ± SD. **p < 0.01, ***p < 0.001; Student’s *t* test.

The finding of decreased muscle mass in *FoxM1-cKO* mice prompted us to examine the abundance of SCs in the skeletal muscle. Since SCs have definite cell surface markers (defined as CD45^−^Sca1^−^CD11b^−^CD31^−^CD34^+^α7-integrin^+^)^31^, we utilized flow cytometry to analyze the SC pool. FoxM1 deletion had no obvious effect on the abundance of SCs in 2-month-old mice (Supplementary Fig. 2a) but considerably reduced SCs abundance in 8-month-old mice (Fig. 2a, b). Immunostaining revealed considerably fewer numbers of Pax7^+^ SCs per fiber in 8-month-old *FoxM1*-*cKO* mice than in their control littermates (Fig. 2c, d). These data suggested that loss of FoxM1 impaired SC maintenance.

We analyzed the cell cycle of SCs and observed a significantly increased frequency of quiescent SCs in *FoxM1-cKO* mice (Fig. 2e, f). We also found a decreased percentage of S phase SCs in *FoxM1*-*cKO* mice compared with control mice (Fig. 2g, h). In vitro analysis showed that deletion of *FoxM1* inhibited the incorporation of bromodeoxyuridine (BrdU) and expansion of SCs (Supplementary Fig. 2b, c and Fig. 2i). Loss of FoxM1 may result in cell apoptosis. Deletion of FoxM1 resulted in a slight increase in apoptosis (Fig. 2j). The differentiation potential of SCs is vital for muscle regeneration. However, we found that the loss of FoxM1 had no obvious effect on SC differentiation (Supplementary Fig. 2d–f). Together, these data suggested that FoxM1 was essential for SC maintenance by regulating the proliferation and survival of SCs.

### Loss of FoxM1 results in activation of wnt/β-catenin signaling in SCs

**Fig. 3:**
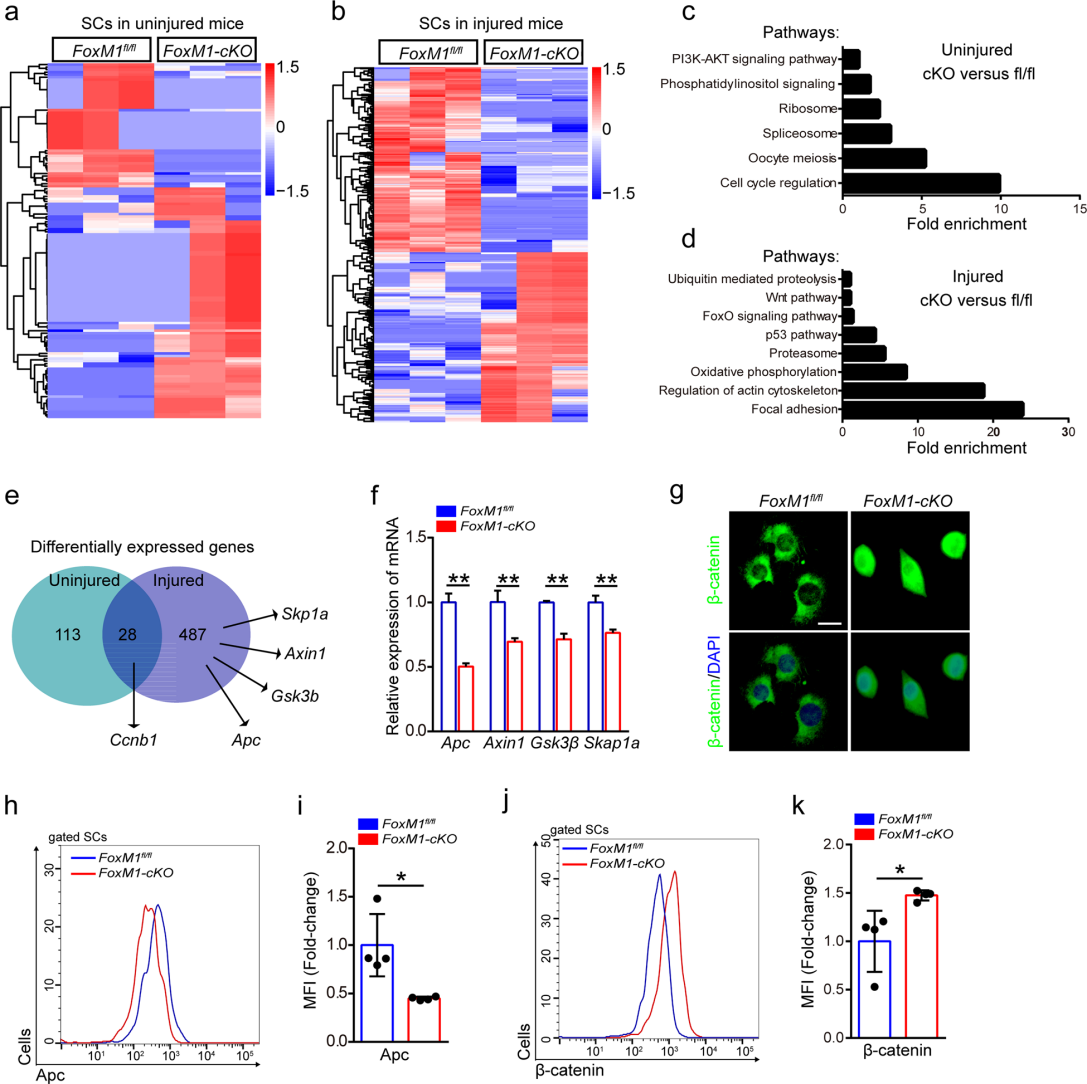
Loss of FoxM1 results in activation of wnt/β-catenin signaling in SCs. **a**, **b** Heatmaps showing the differential expressed protein-coding genes of SCs in uninjured mice (**a**) or injured mice (**b**) (*n* = 3). SCs were isolated from 2-month-old *FoxM1*-*cKO* mice that were uninjured or 48 h post-injury by flow cytometry. The sorted SCs were conducted by RNA-Sequencing analysis. **c**, **d** Enrichment of differentially expressed genes into pathways of SCs in uninjured mice (**c**) or injured mice (**d**). **e** Numbers of dysregulated protein-coding genes (data from RNA-Sequencing) in SCs. **f** SCs were sorted from *FoxM1*^*fl/fl*^ and *FoxM1-cKO* mice (2 months of age) at 48 h post-injury. Total RNA of sorted SCs was extracted and the expression of the indicated genes was assayed by qPCR (*n* = 4). **g** SCs were isolated from *FoxM1*^*fl/fl*^ and *FoxM1-cKO* mice (2 months of age) and cultured in vitro. Immunofluorescence staining of β-catenin in SCs (*n* = 3). Nuclei were stained with DAPI. Scale bars, 20 μm. **h** The mononuclear cells were isolated from the skeletal muscles of mice (2 months of age) at 48 h post-injury and were stained with SC markers and Apc fluorescent antibody. The intensity of Apc was analyzed by flow cytometry. **i** The mean fluorescence intensity (MFI) analysis of Apc in SCs (*n* = 4). **j** The mononuclear cells were isolated from the skeletal muscles of mice (2 months of age) at 48 h post-injury and were stained with SC markers and β-catenin fluorescent antibody. The intensity of β-catenin was analyzed by flow cytometry. **k** The MFI analysis of β-catenin in SCs (*n* = 4). Error bars represent the means ± SD. *p < 0.05, **p < 0.01; Student’s *t* test.

We performed global gene expression profiling of SCs isolated from 2-month-old *FoxM1*-*cKO* mice that were uninjured or 48 h post-injury. We found that 141 genes had over twofold differential expression in *FoxM1-cKO* SCs relative to their expression in *FoxM1*^*fl/fl*^ SCs in uninjured mice, In injured mice, 515 genes were differentially expressed (Fig. 3a, b). Notably, cell cycle regulation and the ribosome pathway were the most commonly modified cellular function pathways in *FoxM1-cKO* SCs under homeostatic status, and p53, Foxo, and wnt pathways were enriched in injured mice (Fig. 3c, d). The differential expression of 28 genes, including the cell cycle regulator, Ccnb1, occurred in both the homeostatic and injured conditions (Fig. 3e). These results revealed that FoxM1 promoted cell cycling of SCs at the molecular level.

We also found genes of wnt signaling pathway that displayed downregulated expression in SCs in injured mice. These genes included Apc, Gsk3β, Axin1, and Skp1a (Fig. 3e). Wnt signaling plays an essential role during embryonic muscle development and in the maintenance of skeletal muscle homeostasis^32,33^. Quantitative real-time polymerase chain reaction (qPCR) results confirmed the significant downregulation of the aforementioned genes (Fig. 3f). The expression of Apc showed similar pattern of change in SCs during muscle regeneration (Supplementary Fig. 2g, h). Moreover, the protein level of Apc in SCs in vivo was reduced in *FoxM1-cKO* mice compared with control mice (Fig. 3h, i). The cultured FoxM1-deficient SCs displayed increased total and nuclear β-catenin proteins compared with control SCs in vitro (Fig. 3g). Consistently, the protein level of β-catenin in SCs in vivo was upregulated in FoxM1-deficient mice compared with control mice (Fig. 3j, k). The expression of Tgf-β2 and Tgf-β3 was also upregulated in FoxM1-deficient SCs in the injured condition (Supplementary Fig. 2i, j). Together, these data suggested that loss of FoxM1 resulted in activation of wnt/β-catenin signaling in SCs.

### FoxM1 preserves muscle regeneration by transcriptionally regulating Apc in SCs

**Fig. 4:**
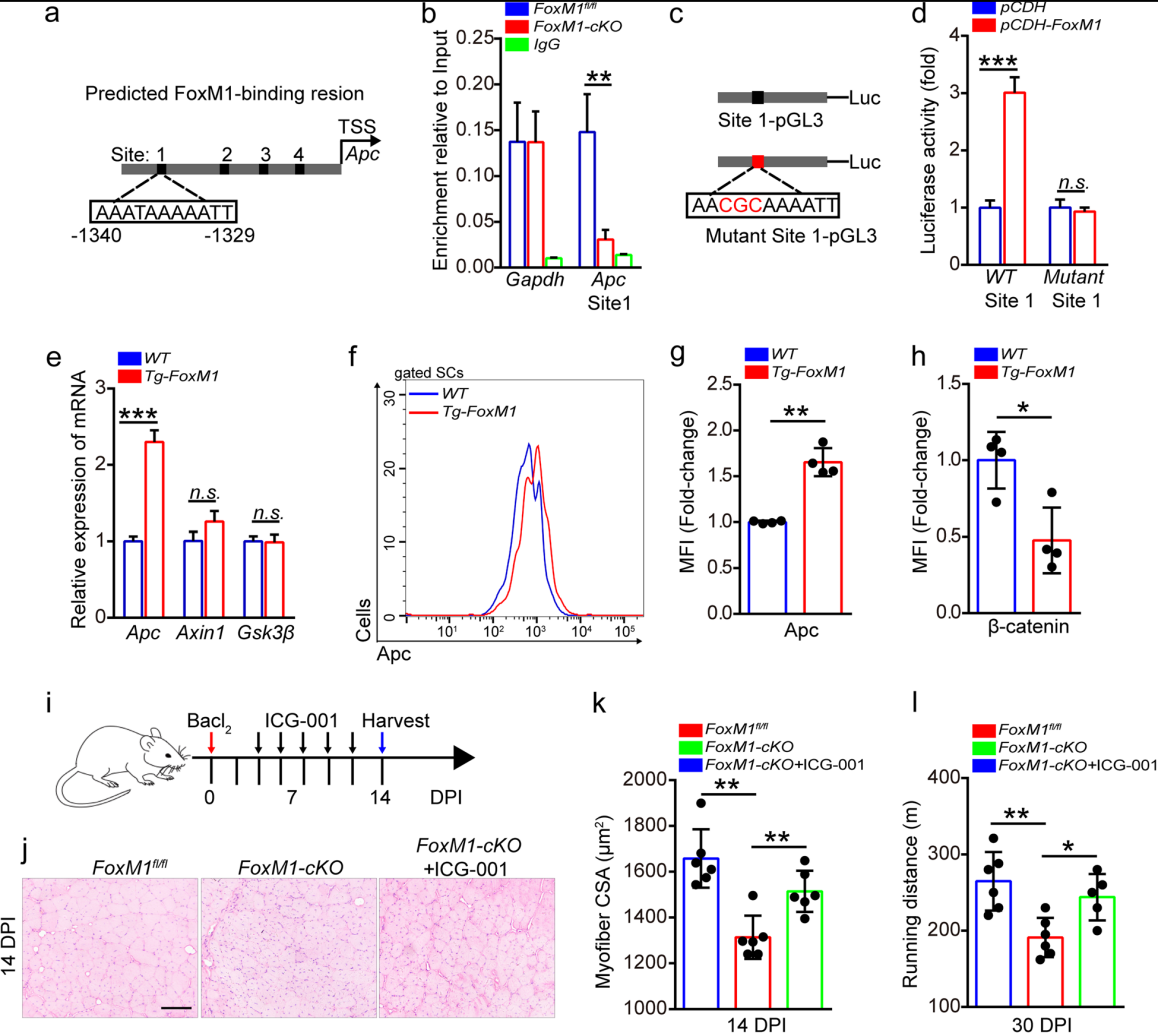
FoxM1 preserves muscle regeneration by transcriptionally regulating Apc in SCs. **a** The upstream promoter regions of mouse Apc gene, including predicted FoxM1-binding sites (black). **b** Quantitative PCR analysis of the FoxM1 binding to promoter regions of Apc in SCs. Enrichment relative to the input was shown (*n* = 4). SCs were isolated from *FoxM1*^*fl/fl*^ and *FoxM1-cKO* mice (2 months of age) and cultured in vitro. IgG served as a negative control and the binding of RNA polymerase II to GAPDH promoter served as a positive control. **c** Wild-type and mutant (mut) Apc promoter luciferase (luc) constructs. **d** Luciferase activity in 293T cells transfected with a luciferase reporter vector (PGL3) with the WT or mutant promoter of Apc, as well as pCDH and pCDH-FoxM1. Results are presented relative to those of cells transfected with the luciferase construct and empty vector (pCDH) (*n* = 3). **e** SCs were sorted from *WT* and *Tg-FoxM1* mice (2 months of age) at 48 h post-injury. Total RNA of sorted SCs was extracted and the expression of indicated genes was assayed by qPCR (*n* = 4). **f** The mononuclear cells were stained with SC markers and Apc fluorescent antibody, which were isolated from the skeletal muscles of mice (2 months of age) at 48 h post-injury. The intensity of Apc was analyzed by flow cytometry. **g** The MFI analysis of Apc in SCs (*n* = 4). **h** The MFI analysis of β-catenin in SCs (*n* = 4). The mononuclear cells were isolated from the skeletal muscles of mice (2 months of age) at 48 h post-injury and were stained with SC markers and β-catenin fluorescent antibody. **i** A schematic illustration showing the experimental design for the treatment of ICG-001 in injured mice (2 months of age). **j** HE staining of the TA muscle cross-sections of mice at 14 DPI. Scale bar, 100 μm. **k** Average CSA of TA muscles of mice at 14 DPI (*n* = 6). **l** The maximum running distance of mice, measured by a treadmill at 30 DPI (*n* = 6). Error bars represent the means ± SD. *p < 0.05, **p < 0.01, ***p < 0.001, n.s. no significance; Student’s *t* test or one-way ANOVA.

To determine whether FoxM1 regulates Apc through direct transcriptional activation, we searched for the consensus FoxM1-binding site (C/T)AAA(C/T)AA in the proximal promoter regions of Apc. A chromatin immunoprecipitation (ChIP) assay of SCs revealed the direct binding of FoxM1 to site 1 in the Apc promoter regions (Fig. 4a, b). The luciferase activity of a construct containing the wild-type (WT)-binding site showed apparent increases with FoxM1 expression. However, the construct containing the mutants did not (Fig. 4c, d). We generated mice with FoxM1 overexpression in SCs (designated as “*Tg-FoxM1*” mice here). The strategy efficiently promoted the overexpression of FoxM1 in SCs (Supplementary Fig. 3a–c). Inspiringly, FoxM1 overexpression significantly upregulated the expression of Apc in SCs (Fig. 4e), as well as the protein level of Apc (Fig. 4f, g). Subsequently, the total protein level of β-catenin was strikingly reduced (Fig. 4h). Loss- and gain-of-function analyses demonstrated a novel mechanism in which FoxM1 repressed wnt/β-catenin signaling by transcriptionally regulating Apc in SCs.

To determine whether the downregulated expression of Apc hindered muscle regeneration of *FoxM1-cKO* mice by the Apc-β-catenin axis, the mice were administered ICG-001, a small molecule inhibitor of wnt/β-catenin^34^. Treatment with ICG-001 partially rescued the regeneration capacity of *FoxM1*-*cKO* SCs, as shown by larger cross-sectional area (CSA), normal regenerative myofiber, and enhanced exercise performance (Fig. 4i–l). These data suggested that the regeneration defect of FoxM1-deficient SCs was partially due to the hyperactivation of wnt/β-catenin signaling resulting from reduced Apc expression.

### FoxM1 overexpression promotes muscle hypertrophy but eventually results in muscle atrophy

**Fig. 5:**
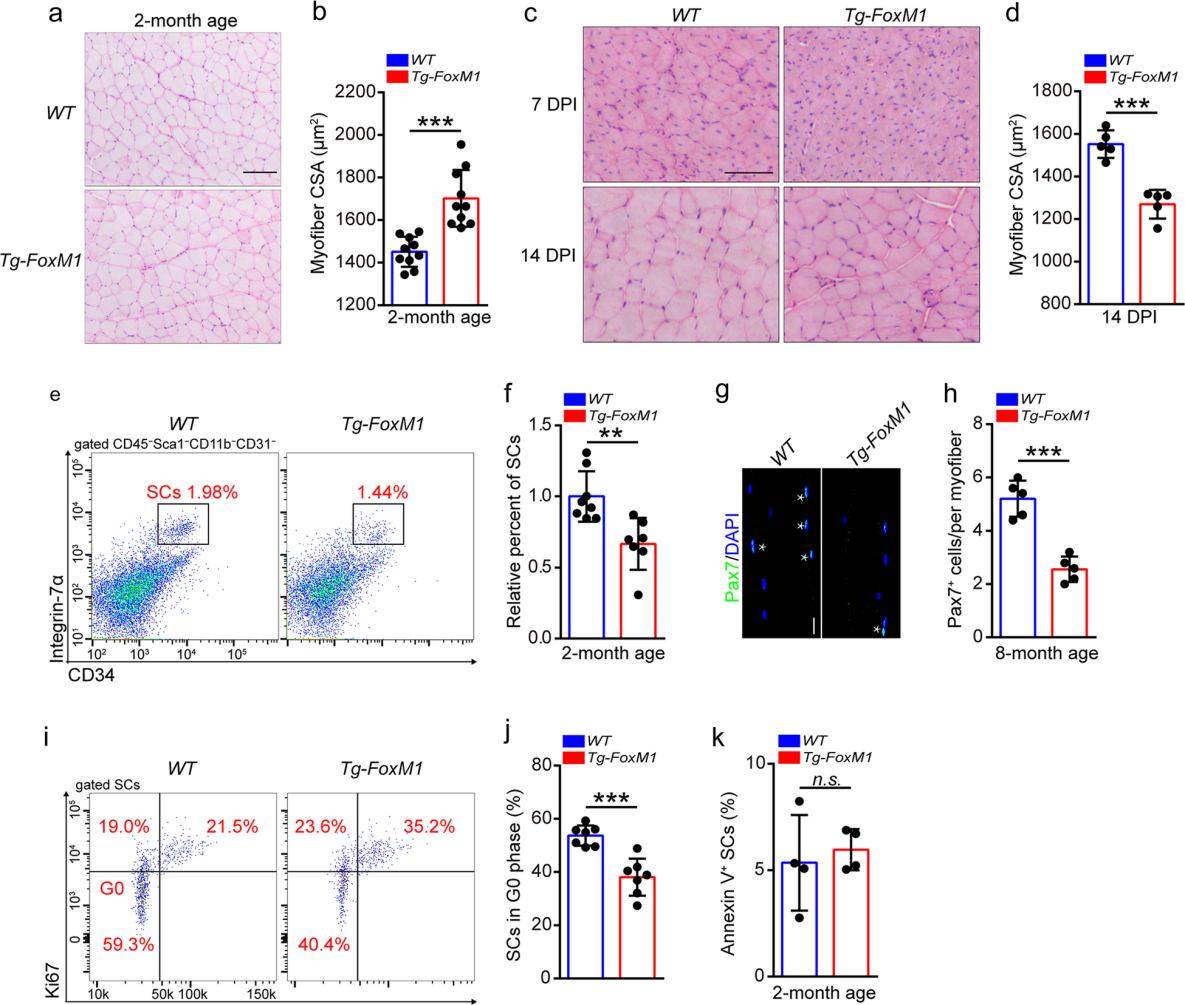
FoxM1 overexpression promotes muscle hypertrophy but eventually results in muscle atrophy. **a** HE staining of the TA muscle cross-sections in FoxM1 overexpression mice compared with control mice at 2 months of age. Scale bar, 100 μm. **b** Average CSA of TA of *Tg-FoxM1* and *WT* mice (2 months of age) (*n* = 10). **c** HE staining of the TA muscle cross-sections of *Tg-FoxM1* and *WT* mice (2 months of age) at 7 DPI and 14 DPI. Scale bar, 100 μm. **d** Average CSA of TA muscle of mice (2 months of age) at 14 DPI (*n* = 5). **e** The mononuclear cells were isolated from skeletal muscles of mice (2 months of age) and were stained with SC markers. The population of SCs was analyzed by flow cytometry. **f** Quantification of the relative percentage of SCs per total mononuclear cells isolated from the skeletal muscles of mice at 2 months of age (*n* = 8). **g** Skeletal muscles were harvested from mice (8 months of age) and digested as myofibers. Representative fluorescent images of fresh myofibers immunostained with Pax7 antibody (green) and DAPI (blue). Scale bars, 50 μm. **h** Quantification of the numbers of Pax7^+^ SCs per myofiber of mice at 8 months of age (*n* = 5, 20 myofibers per mouse). **i** The mononuclear cells were isolated from the skeletal muscles of mice (2 months of age) and were stained with cell surface markers, DAPI and Ki67. The quiescent percentage of SCs was analyzed by flow cytometry. **j** Quantification of the percentage of G0 phase in SCs of mice (2 months of age) (*n* = 6). **k** The mononuclear cells were isolated from the skeletal muscles of mice (2 months of age) and were stained with SC markers, as well as Annexin-V and DAPI. The percentage of apoptotic SCs (Annexin-V^+^ SCs) was analyzed by flow cytometry (*n* = 4). Error bars represent the means **±** SD. *p < 0.05, **p < 0.01, ***p < 0.001, n.s. no significance; Student’s *t* test.

We observed obvious muscle hypertrophy in *Tg-FoxM1* mice compared with 2-month-old *WT* mice (Supplementary Fig. 3d and Fig. 5a, b). However, FoxM1 overexpression in SCs eventually resulted in striking muscle atrophy at 8 months of age (Supplementary Fig. 3e–i). Moreover, the regenerative myofibers of 2-month-old *Tg-FoxM1* mice were significantly smaller than those in *WT* mice (Fig. 5c, d). These data indicated that FoxM1 overexpression in SCs promoted myogenesis at an early age but impaired muscle regeneration under stress and muscle development of mice with increasing age.

The transition of muscle hypertrophy to atrophy implied the exhaustion of SC pool in *Tg-FoxM1* mice. We observed a decrease in the SC abundance in *Tg-FoxM1* mice compared with *WT* mice at 2 months of age (Fig. 5e, f). The difference was more striking at 8 months of age and at 30 DPI (Supplementary Fig. 3j–l). The numbers of Pax7^+^ SCs in single fibers in *Tg-FoxM1* mice were also significantly reduced compared to *WT* mice at 8 months of age (Fig. 5g, h). These data suggested that FoxM1 overexpression resulted in SC exhaustion, which helped explain the eventual muscle atrophy and the impaired muscle regeneration in *Tg-FoxM1* mice.

### FoxM1 promotes cell cycling of SCs by transcriptionally regulating Ccnb1

FoxM1 overexpression promoted the entry of quiescent SCs into the cell cycle (Fig. 5i, j and Supplementary Fig. 3m, n). Flow cytometric analysis revealed that FoxM1 overexpression had no obvious effect on apoptosis of SCs (Fig. 5k). FoxM1 reportedly regulates a subset of G1–G2 and G2–M transition genes. Ccnb1 showed downregulated expression in FoxM1-deficient SCs compared with control SCs (Supplementary Fig. 4a). FoxM1 overexpression significantly upregulated the expression of Ccnb1 in SCs (Supplementary Fig. 4a). The expression of Ccnb1 was strikingly induced by muscle injury (Supplementary Fig. 4b). Moreover, the protein level of Ccnb1 in SCs showed a converse change in *FoxM1-cKO* and *Tg-FoxM1* compared to control mice in vivo (Supplementary Fig. 4c, d) and in vitro (Supplementary Fig. 4e). ChIP and luciferase reporter assays demonstrated that FoxM1 transcriptionally regulated Ccnb1 expression (Supplementary Fig. 4f–h).

Ccnb1 complexed with Cdk1 is one of the key mitotic kinases, and Cdk1/Ccnb1 governs the entry into mitosis from the G2 phase of the cell cycle^35,36^. We cultured SCs of FoxM1-cKO mice in vitro and enforced Ccnb1 expression in these SCs. Enforced expression of Ccnb1 partially rescued BrdU incorporation in FoxM1-deficient SCs (Supplementary Fig. 4i, j). SCs cultured in vitro were treated with RO-3306, an inhibitor of Cdk1/Ccnb1^37^. RO-3306 treatment in vitro abolished the increased BrdU incorporation of FoxM1-overexpressing SCs (Supplementary Fig. 4i, k). Although the injection of RO-3306 in vivo affected all muscle cells, RO-3306 treatment efficiently restored the frequency of quiescent SCs in *Tg-FoxM1* mice and rescued the decreased SC pool (Supplementary Fig. 5a–d). Treatment with RO-3306 had no obvious effect on the numbers of Pax7^+^ SCs but considerably recovered the decreased SC pool in *Tg-FoxM1* mice (Supplementary Fig. 5e). The treatment of RO-3306 also abolished muscle hypertrophy in 2-month-old *Tg-FoxM1* mice (Supplementary Fig. 5f, g). Since Cdk1 mediates the phosphorylation of FoxM1, it is possible that the inhibition of Cdk1/Ccnb1 eventually affected the phosphorylation of FoxM1 and its transcriptional activity. Together, these data demonstrated that FoxM1 promoted cell cycling of SCs partially by transcriptionally regulating Ccnb1.

### Cdh1 prevents accumulation of FoxM1 protein by ubiquitylation

**Fig. 6:**
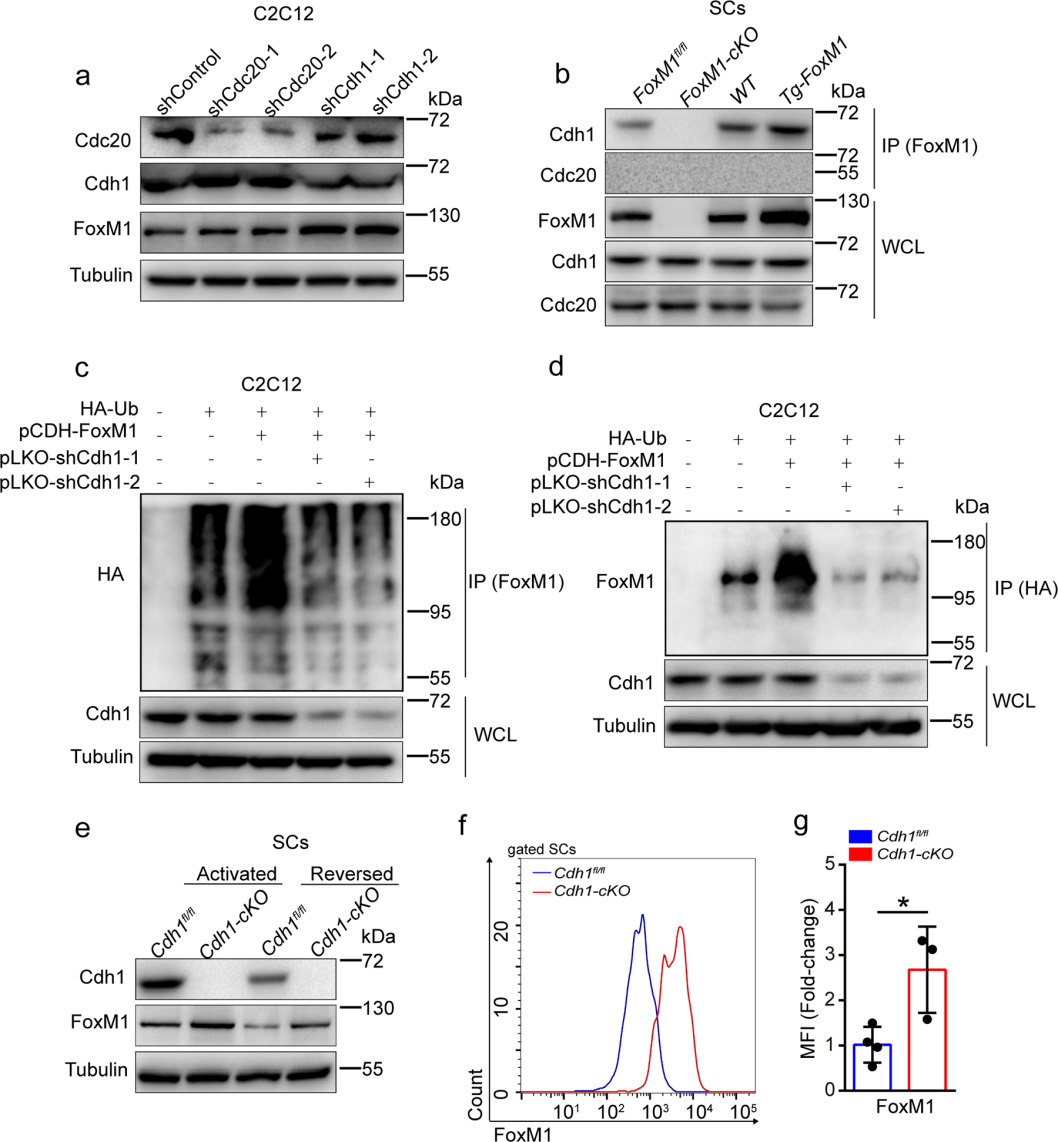
Cdh1 prevents accumulation of FoxM1 protein by ubiquitylation. **a** Cdc20 and Cdh1 were knocked down in proliferative C2C12 using independent shRNAs (pLKO construct). Levels of Cdc20, Cdh1, FoxM1, and Tubulin (loading control) were determined by western blot analysis (*n* = 3). **b** SCs were isolated from FoxM1 deletion or overexpression mice and were cultured in vitro. Cdh1 proteins were immunoprecipitated from the cell lysates and analyzed by western blot (*n* = 3). WCL whole-cell lysates. **c**, **d** HA-ubiquitin (HA-Ub, ubiquitin expression construct with HA tag) plasmid was transfected with the indicated plasmids (FoxM1 overexpression plasmid, pCDH-FoxM1; Cdh1 knockdown plasmid, pLKO-shCdh1-1, pLKO-shCdh1-2) in proliferative C2C12 cells; 8 h after 10 μM MG132 treatment, HA-Ubiquitin (**c**) or FoxM1 (**d**) proteins were immunoprecipitated from cell lysates and analyzed by western blot (*n* = 4). WCL whole-cell lysates. **e** SCs were isolated from *Cdh1*^*fl/fl*^ and *Cdh1*^*fl/fl*^*Pax7-Cre* (called *Cdh1-cKO* here) and were cultured in growth medium (activated state) or serum-free medium (reversed state) in vitro. Whole-cell lysates were subjected to western blot analysis using the indicated antibodies (*n* = 3). **f** The mononuclear cells were isolated from skeletal muscles of mice (2 months of age) and were stained with SC markers and FoxM1 fluorescent antibody. The intensity of FoxM1 was analyzed by flow cytometry. **g** The MFI analysis of FoxM1 in SCs (*n* = 3–4). Error bars represent the means ± SD. *p < 0.05; Student’s *t* test.

The APC/C is a multisubunit E3 ubiquitin ligase complex that regulates cyclic degradation of cell cycle regulator via Cdh1 or Cdc20 adaptor proteins^38^. We knocked down the expression of Cdh1 or Cdc20 by two independent short hairpin RNAs (shRNAs) in C2C12 cells. Reduction of Cdh1, but not Cdc20, resulted in increased FoxM1 protein in proliferative C2C12 cells (Fig. 6a). Knockdown or knockout of FoxM1 had no obvious effect on the expression of Cdh1 and Cdc20 in C2C12 or SCs (Supplementary Fig. 6a, b). Furthermore, co-immunoprecipitation assay revealed the direct interaction between FoxM1 and Cdh1, but not Cdc20, in SCs (Fig. 6b and Supplementary Fig. 6c). APC/C^Cdh1^ induces the degradation of its substrates through ubiquitin-dependent proteolysis^39^. As expected, knockdown of Cdh1 reduced poly-ubiquitylation of FoxM1 in C2C12 cells (Fig. 6c, d). These data suggested that APC/C^Cdh1^ promoted FoxM1 degradation through the ubiquitin–proteasome pathway in C2C12 cells in vitro.

We generated the conditional knockout mouse *Cdh1*^*fl/fl*^*Pax7-Cre* (designated as *Cdh1-cKO*) and its control (*Cdh1*^*fl/fl*^) (Supplementary Fig. 6e, f). The FoxM1 protein level in *Cdh1-cKO* SCs was increased compared to *Cdh1*^*fl/fl*^ SCs in vitro (Fig. 6e). The mRNA and protein levels of Cdh1 showed peaks at 7 DPI (Supplementary Fig. 6d). The protein level of FoxM1 in SCs was strikingly elevated in *Cdh1-cKO* mice (Fig. 6f, g). Together, these data suggested that Cdh1 prevented accumulation of FoxM1 protein by ubiquitylation.

### Loss of Cdh1 results in the loss of SC pool

**Fig. 7:**
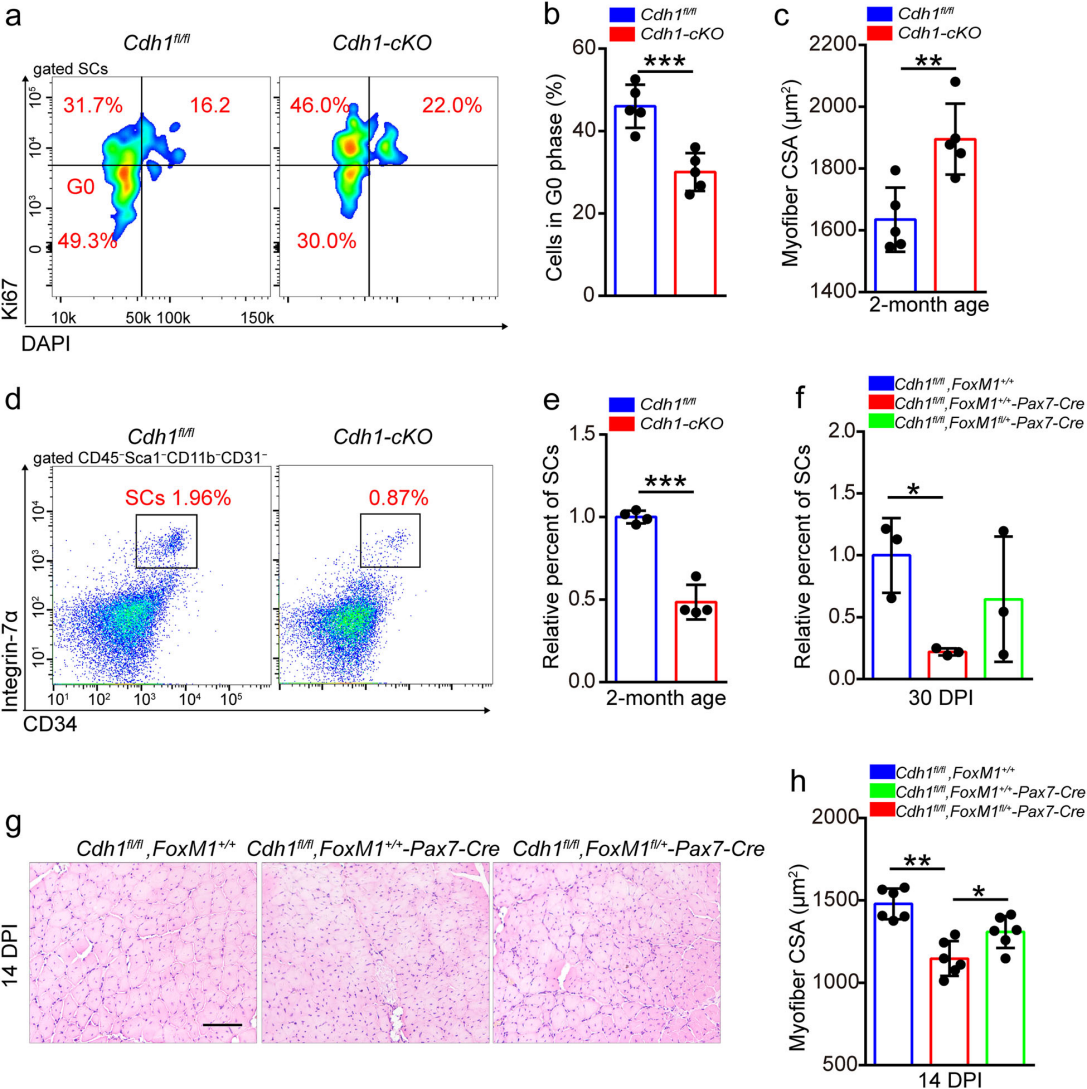
Loss of Cdh1 results in the loss of SC pool. **a** The mononuclear cells were isolated from the skeletal muscles of mice (2 months of age) and were stained with cell surface markers, DAPI and Ki67. The quiescent percentage of SCs was analyzed by flow cytometry. **b** Quantification of the percentage of G0 phase in SCs of mice (2 months of age) (*n* = 5). **c** Average CSA of TA in *Cdh1*^*fl/fl*^ and *Cdh1-cKO* mice (2 months of age) (*n* = 5). **d** The mononuclear cells were isolated from skeletal muscles of mice (2 months of age), and were stained with SC markers. The population of SCs was analyzed by flow cytometry. **e** Quantification of the relative percentage of SCs per total mononuclear cells isolated from the skeletal muscles of mice at 2 months of age (*n* = 4). **f** Quantification of the relative percentage of SCs per total mononuclear cells isolated from the skeletal muscles of mice (2 months of age) at 30 DPI (*n* = 3). **g** Mice were induced with serial muscle injuries by BaCl_2_ injection. HE staining of the TA muscle cross-sections of mice (2 months of age) at 14 DPI of the second injury. Scale bar, 100 μm. **h** Average CSA of TA muscles of mice (2 months of age) at 14 DPI of the second injury (*n* = 6). Error bars represent the means ± SD. *p < 0.05, **p < 0.01, ***p < 0.001; Student’s *t* test or one-way ANOVA.

Knockout of Cdh1 evidently decreased the percentage of quiescent SCs (Fig. 7a, b) and depleted the SC pool at 2 months of age (Fig. 7d, e). Moreover, we detected muscle hypertrophy in *Cdh1-cKO* mice compared to *Cdh1*^*fl/fl*^ mice at the same age (Fig. 7c). We generated *Cdh1*^*fl/fl*^, *FoxM1*^*fl/+*^, and *Pax7-Cre* mice to test whether haploinsufficiency of FoxM1 could rescue the exhaustion of SCs in *Cdh1-cKO* mice. Deletion of Cdh1 resulted in a remarkable reduction of SCs at 30 DPI, while haploinsufficiency of FoxM1 partially restored the quantity of SCs (Fig. 7f). Consistently, serial injuries resulted in a severe regeneration defect in *Cdh1-cKO* mice, but haploinsufficiency of FoxM1 improved the muscle regeneration of Cdh1-deficient mice (Fig. 7g, h). Together, these data suggested that Cdh1 prevented FoxM1 accumulation to maintain muscle homeostasis.

## Discussion

FoxM1 is a well-established regulatory transcriptional factor for cell cycle progression^40,41^. Recently, increasing evidence has suggested the pleiotropic role of FoxM1 in stem cells^19,20^. In this study, we identified previously unrecognized roles of FoxM1 in muscle homeostasis and SC maintenance. We found that FoxM1 promoted cell cycling of SCs partially by transcriptionally regulating Ccnb1. FoxM1 also preserved regenerative potential of SCs by transcriptionally regulating Apc to inhibit wnt/β-catenin signaling. The degradation of FoxM1protein is regulated by APC/C^Cdh1^ via ubiquitylation.

FoxM1 acts as a cell cycle regulator and induces cell proliferation^42–44^. In this study, we found that loss of FoxM1 in SCs resulted in decreased SC pool with age, and the percentage of quiescent SCs considerably reduced. However, overexpression of FoxM1 evidently promoted cell cycle entry of SCs and enhanced proliferative capacity of SCs. It is possible that the quiescence exit and the increased proliferation of SCs in *Tg-FoxM1* mice in combination results in SC exhaustion with age or under stress. Muscle development relies on proliferation and differentiation potential of SCs. We found that loss of FoxM1 had no obvious effect on SC differentiation. Thus the eventual muscle atrophy observed in *FoxM1-cKO* and *Tg-FoxM1* mice could be attributed to the altered cell cycle of SCs and the reduced SC pool.

FoxM1 has been reported to regulate a subset of G1–G2 and G2–M transition genes in stage- and context-dependent manners^42–44^. Ccnb1 is a transcriptional target of FoxM1 in human hepatocellular carcinoma cells^45^ and human hTERT RPE-1 cells^46^. Ccnb1 binds to its partner Cdk1, and Cdk1/Ccnb1 is crucial for G2–M transition of the cell cycle^47^. Cdk1 can directly phosphorylate FoxM1 to increase its transcriptional activity^46,48^. Presently, FoxM1 directly regulated Ccnb1 expression by binding to its proximal promoter region. Inhibition of Cdk1/Ccnb1 partially rescued the loss of quiescent SCs in FoxM1-overexpressing mice. Thus we consolidated the reported mechanisms of FoxM1 transcriptionally regulating Ccnb1. We also found that ribosome-related genes were downregulated in FoxM1-deficient SCs, which helped explain the phenomenon that the enforced expression of Ccnb1 could not completely recover proliferation capacity of FoxM1-deficient SCs. Our data suggest that FoxM1 maintains the SC pool partially by transcriptionally regulating Ccnb1. Whether FoxM1 promotes the proliferation of SCs by ribosome biogenesis requires further studies.

Besides the effect of FoxM1 on SC cell cycling, we found that loss of FoxM1 severely impaired the regeneration potential of SCs. *FoxM1-cKO* mice showed largely unrepaired muscle regions compared to *FoxM1*^*fl/fl*^ mice, which could not be simply attributed to the altered cell cycle of SCs. Through RNA-Sequencing, we found that knockdown of FoxM1 resulted in downregulation of genes related to the wnt pathway, including Apc, Axin1, and Gsk3β. Importantly, muscle regeneration required inhibited wnt/β-catenin signaling and Apc interaction with Axin, Cki, and Gsk3β to form a cytoplasmic destruction complex to phosphorylate β-catenin, leading to destruction of β-catenin via the ubiquitylation pathway^27^. The loss of Apc in SCs does not affect skeletal muscle tissue integrity but results in severe muscle regeneration defects^33^. β-Catenin constitutively active mice are not affected with respect to the number of SCs in the homeostatic condition. However, these mice display muscle regeneration defects with aberrant myofiber morphology and atrophy, which is mainly attributed to hyperactive Tgf-β signaling^28^. The phenotype of muscle regeneration in *FoxM1-cKO* mice was similar to that in Apc-deficient and β-catenin constitutively active mice. Furthermore, the protein level of β-catenin in FoxM1-deficient SCs was considerably increased following injury, raising a possibility that FoxM1 can inhibit wnt/β-catenin signaling to preserve regenerative potential of SCs.

The crosstalk of FoxM1 and wnt/Apc/β-catenin signaling is complicated. It has been reported that Gsk3 phosphorylates FoxM1 on serine 474, which induces FoxM1 ubiquitylation and subsequent degradation in LN229 and U87 cells^25^. Wnt3a increases the level and nuclear translocation of FoxM1, which binds directly to β-catenin and enhances β-catenin nuclear localization in 293T cells^26^. Presently, FoxM1 transcriptionally regulated the expression of Apc to restrict the activation of β-catenin signaling in SCs under stress. We speculate that the differing observations of our study may be attributed to the differences in cell types. Inhibition of wnt/β-catenin signaling by ICG-001 efficiently rescued the aberrant myofiber morphology and atrophy in *FoxM1-cKO* mice after injury. These findings expand our insights into the interwoven intercommunication between FoxM1 and wnt/Apc/β-catenin signaling and reveal the protective function of FoxM1 in muscle regeneration. This may be an adaptive strategy for SCs upon injury. Whether the FoxM1–Apc–β-catenin axis exists in other stem cells is unclear and warrants further investigation.

FoxM1 was required for SC proliferation and muscle regeneration, but excessive FoxM1 resulted in SC exhaustion. The expression of FoxM1 in SCs during muscle regeneration also showed a process of upregulation to downregulation. The protein level of FoxM1 was evidently increased in activated C2C12 compared to quiescent cells^21^ and notably decreased when returning to quiescent state (for C2C12 and SCs). Thus a regulatory network may precisely control the expression of FoxM1 at the transcriptional and epigenetic level. Proteolysis by the ubiquitin–proteasome system is a major mechanism involved in myofibrillar protein degradation. FoxM1 interacts with the complex of E3 ligase APC/C and its adaptor, Cdh1. Cdh1 stimulated degradation of FoxM1 protein in HeLa^49^, U2OS^17^, and MCF-7 cells^50^. Moreover, Fbxl2 (a component of Skp-Cullin-F box ubiquitin E3 ligase)^51^ and VprBP (a component of cullin 4-based E3 ubiquitin ligase)^52^ have been reported to regulate the degradation of FoxM1 in different cells. Here we discovered that FoxM1 directly interacted with Cdh1, rather than the adaptor Cdc20. Knockdown or knockout of Cdh1 resulted in the accumulation of FoxM1 protein in SCs. Cdh1 was required for FoxM1 ubiquitylation and subsequent degradation. The findings expand our knowledge of the relationship between Cdh1 and FoxM1 in stem cells and demonstrate that APC/C^Cdh1^ mediates ubiquitylation and degradation of FoxM1 protein in SCs.

Cdh1 has been implicated in regulating cell cycle entry. Presently, deletion of Cdh1 resulted in decreased quiescent SCs and SC exhaustion. This phenotype is similar to that of FoxM1-deficient SCs. The haploinsufficiency of FoxM1 partially recovered the maintenance of SCs after injury and rescued the muscle regeneration of Cdh1-cKO mice, implying that FoxM1 was one of the key downstream targets of Cdh1 in vivo. The accumulation of ubiquitinated protein increased at 3 DPI, indicating that muscle regeneration is associated with the activation of machinery involved in protein degradation^12^. We observed that knockout of Cdh1 in SCs resulted in impaired muscle regeneration, indicating the important function of ubiquitin–proteasome system in muscle regeneration. Whether Cdh1 regulates SCs maintenance and muscle development with age requires further investigation.

In summary, the data presented here demonstrate that FoxM1 was critical for muscle development and regeneration. FoxM1 regulated cell cycle entry of SCs to meet the needs of myogenesis and muscle development, partially via Ccnb1. Upon injury, FoxM1 transcriptionally regulated Apc expression to repress wnt/β-catenin signaling. The ubiquitylation and degradation of FoxM1 protein in SCs was promoted by APC/C^Cdh1^. These findings uncover an Cdh1–FoxM1–Apc axis that regulates muscle development and regeneration. Whether this axis is associated with muscle diseases and whether it could be potential intervention targets warrants further investigations.

## Methods

### Mice and drug treatment

The mice used had a C57BL/6J background. The *FoxM1*^*fl/+*^ mice carry a *FoxM1* locus in which exons 4–7 were flanked by 2 *loxp* sites, while *Cdh1*^*fl/+*^ mice carry a *Cdh1* locus in which exons 2–14 were flanked by 2 *loxp* sites (Cyagen, China). The *FoxM1*^*fl/fl*^ mice and *Cdh1*^*fl/fl*^ was crossed with *Pax7-Cre* mice to generate *FoxM1*^*fl/fl*^*-Pax7-Cre* SC-specific knockout mice (*FoxM1-cKO*) and *Cdh1*^*fl/fl*^*-Pax7-Cre* SC-specific knockout mice (*Cdh1-cKO*). For SC-specific overexpression of *FoxM1*, we generated a bacterial artificial chromosome (BAC) transgenic mouse lines, in which a “*FoxM1* cds—rBGpA” cassette was inserted into the BAC RP23-267J16 following the start codon (ATG) in exon 1 of *Pax7* gene (*Tg-FoxM1*). All compound genetically engineered mice were a result of breeding of the above mice followed by appropriate PCR-based genotyping with specific primers (Table S1). For drug treatments, ICG-001 (20 mg/kg, 3 injections/week, Selleck) and RO-3306 (4 mg/kg, 4 injections/week, Selleck) were intraperitoneally injected into mice for 2 and 5 weeks, respectively. All the mice experiments were approved by the Animal Committee of the Institute of Zoology, Third Military Medical University.

### Histology and morphometric analysis

TA muscles harvested and were fixed for 12 h using 4% paraformaldehyde and subsequently transferred to 20% sucrose overnight for dehydration. For the assessment of muscle morphology, 10-μm-thick transverse sections of TA muscle were subjected to hematoxylin and eosin staining. For quantitative analysis, the CSA was performed using the AxioVision software (Zeiss).

### Skeletal muscle injury

To induce injury, 50 μl 1.2% BaCl_2_ in phosphate-buffered saline (PBS) or PBS alone was injected into and along the length of TA and gastrocnemius muscles. The hindlimb muscles or TA muscles were harvested 2–30 days after injury depending on the experiments.

### Treadmill test

Mice were acclimated to moderate treadmill running (10 m/min for 5 min daily) on a treadmill (ZH-PT, Zheng Hua) for 3 consecutive days before the test. Then mice ran on the treadmill at 10 m/min for 5 min, and the speed was increased from 10 to 16 m/min and then maintained constant until exhaustion. Running time and running distance were measured.

### Isolation of single myofibers

Single myofibers were isolated from EDL muscles and digested in Dulbecco’s modified Eagle’s medium (DMEM; catalog number: SH30022.01, Hyclone, GE, USA) with 0.2% collagenase (Sigma-Aldrich, USA) at 37 °C for 90 min. Fibers were liberated by trituration in DMEM medium with Pasteur pipettes.

### SC isolation and culture

To culture SCs in vitro, we obtained SCs using single myofiber cultures. Briefly, single fibers were placed in Matrigel-coated dishes (BD Bioscience, USA) in fiber medium consisting of DMEM (Hyclone) with 20% fetal bovine serum (Hyclone), 1% penicillin/streptomycin (Hyclone), and 1% Chick embryo extract (US biological, USA) at 37 °C with 5% CO_2_. SCs migrated off the myofibers in 3–4 days. The correct enrichment of SCs were validated by fluorescence staining of Pax7. To analyze the growth of SCs, isolated SCs were cultured in fiber medium. For inducing the reversed state of SCs, cells were cultured in serum-free DMEM for 72 h. For differentiation, SCs were cultured in DMEM containing 2% horse serum (Gibco, Thermo Fisher, USA) on Matrigel. For the inhibition of Cdk1/Ccnb1, SCs were treated with 0.5 μM RO-3306 (Selleck) for 24 h before analysis.

### Flow cytometry

SC isolation and purification was performed according to the established methods. Briefly, TA muscle of mice was subjected to 0.2% collagenase (Sigma) for 90 min and then 0.2% dispase (Sigma) for 30 min. The cell suspension was filtered through a 70-μm nylon filter (Falcon), and mononuclear cells were collected and subjected to fluorescence-activated cell sorter (FACS; BD FACSAriaII) using immunostaining with the following: biotin anti-mouse CD45 (Biolegend, 103104), biotin anti-mouse/human CD11b (Biolegend, 101204), biotin anti-mouse CD31 (Biolegend, 102404), biotin anti-mouse Sca1 (Biolegend, 108103), streptavidin-APC/Cy7 (Biolegend, 405208), CD34-Alexa Fluor 647 (Biolegend, 152205), and Integrin α7-FITC (eBioscience, 11-5867-42). For the intracellular staining, the SCs were first stained with the surface marker and then subjected to Fixation/Permeabilization Kit (BD) according to the manufacturer’s instruction. Then the cells were stained with anti-Ki67-PE (Biolegend, 652404) or anti-Cyclin B1-PE (Biolegend, 647903) or stained with anti-Apc (Beyotime, AF2113) or anti-Cdh1 (Proteintech, 16368-1-AP) and incubated with anti-rabbit-FITC (Biolegend, A120-101F).

For BrdU incorporation analysis, SCs cultured in vitro were incubated with 10 μM BrdU (Sigma) for 30 min, washed with PBS, fixed with 70% absolute ethyl alcohol for 6 h, permeabilized with 0.1% Triton X-100 for 10 min, incubated with 2 M HCl for 30 min, and blocked with 2% horse serum for 1 h. Then cells were stained with anti-BrdU-FITC (Biolegend, 364104) for 30 min and analyzed by flow cytometry.

### Immuofluorescence

Myofibers or SCs were fixed with 4% paraformaldehyde for 30 min at room temperature, washed, and then incubated in 0.1% Trixon X-100 for 10 min. Then samples were blocked with 2% horse serum for 1 h and incubated with primary antibodies at 4 °C overnight. The following antibodies were used: Pax7 (Invitrogen, PA1-117, 1:100), MyHC (R&D, MAB4470, 1:50), and β-catenin (Proteintech, 51067-2-AP, 1:200). After the primary antibody incubation, myofibers or SCs were incubated with secondary antibodies conjugated with Alexa Fluor 488 or 647 (Beyotime, 1:500). Finally, the nuclei were staining by 4,6-diamidino-2-phenylindole (5 μg/ml, Sigma) for 10 min, and the fluorescent pictures were captured by confocal microscopy (Olympus).

### Cell proliferation assay

To assess the altered cell phenotypes, we seeded the SCs in 96-well plates with 2000 cells per well in triplicate. At 24, 48, and 72 h post culture, cell proliferation was assessed using the Cell Counting Kit-8 Kit (Beyotime) according to the manufacturer’s instruction.

### Library preparation and RNA-Sequencing

About 800 SCs from mice (homeostatic condition or 48 h-post injury) were isolated by FACS, and the mRNA library was constructed using the QIAseq FX Single Cell RNA Library Kit (QIAGEN) according to the manufacturer’s instruction. Libraries were sequenced by the Illumina HiSeq 2000 platform as 150-bp pair-ended reads. Reads were aligned using bowtie v0.12.9. FPKM estimation was performed with Cufflinks v2.1.1, aligned reads were counted with HTSeq, and differential expression analysis was performed with DESeq2. Differentially expressed genes were selected using a cut-off at a *p* value of <0.05 (false discovery rate adjusted for multiple testing).

### RNA extraction and qPCR

Total cellular RNA was isolated from 3000 SCs immediately after FACS sorting using the Total RNA Isolation Kit (Thermo Fisher) according to the manufacturer’s instructions. cDNA was reverse transcribed using the PrimeScript RT Reagent Kit (Takara) and subjected to real-time PCR with SYBR Green Supermix (Bio-Rad) in an iCycler iQ Real Time PCR Detection System (Bio-rad). All primers are listed in Table S1. All samples were run in triplicate. β-Actin (Actinb) was used as an internal control for mRNA.

### Lentiviral constructs and packaging

To generate the vectors for the expression of FoxM1, CDC20, and CDH1-specific shRNA, we designed the sequence of shRNAs and cloned shRNAs into the vector pLKO.1-puro (primer sequences, Table S1). For generation of the vectors of expression of FoxM1, we amplified transcriptional regions by reverse transcription–PCR and cloned the regions into pCDH-puro (primer sequences, Table S1). The HA-Ubiquitin plasmid was a gift from Dr. Zhigang Li. For plasmid transfection, C2C12 or 293T cells were transfected with the indicated plasmids using polyetherimide (Sigma-Aldrich, USA). For lentivirus production, 293T cells were transfected with the helper plasmid pSPAX2 and pMD2.G. The medium was replaced with fresh medium at 10 h after transfection. The culture supernatants were collected at 48 h after transfection and filtered by 0.22-μm membrane. Virus was stored at −80 °C until use. For lentiviral infection of cells, lentiviral stock was added to C2C12 or SCs with polybrene (8 μg/ml, Sigma-Aldrich, USA). After incubation overnight, supernatants was removed, and cells were washed with PBS and then resuspended in fresh medium with or without puromycin (3 μg/ml, Sigma-Aldrich) to screen stable cell lines.

### Immunoprecipitation and western blot

Cells were prepared using RIPA buffer (Beyotime) containing protease inhibitor mixture (Beyotime). Immnoprecipitates or total cell lysates were analyzed by western blot according to standard procedures. The antibodies used in this study were against: Cdc20 (Proteintech, 10252-1-AP, 1:1000), Cdh1 (Santa, sc-56312 1:1000), FoxM1 (Proteintech, 13147-1-AP, 1:1000), Cyclin B1 (CST, 4138T, 1:1000), Cyclin B2 (Beyotime, AF2509, 1:1000), Apc (Beyotime, AF2113, 1:1000), β-catenin (Proteintech, 51067-2-AP, 1:1000), HA (Proteintech, 66006-2-Ig, 1:10,000), and Tubulin (Beyotime, AF0001, 1:1000).

### ChIP assay

SCs were harvested and used for ChIP assays according to the manufacturer’s instruction of the EZ-ChIP Chromatin Immunoprecipitation Kit (Millipore). In brief, the cells were fixed with 1% formaldehyde for 10 min, and the fixation reaction was quenched with glycine to a final concentration of 125 mM. The cells were lysed and sonicated until the desired lengths were achieved (100–500 bp). Then 5 μg of anti-FoxM1 (Proteintech) or control IgG were used for immunoprecipitation. After elution of DNA from the precipitated immunocomplexes, PCR or qPCR were performed with the specific primers (Table S1).

### Luciferase reporter assay

The corresponding genomic regions that included the putative binding sites was amplified by PCR from WT C57BL/6 mouse genomic DNA (primer sequences, Table S1), then were subsequently cloned into a pGL3-basic luciferase reporter vector. The vectors with mutant-binding sites were generated by site-directed mutagenesis with 3 bases’ mutation (primer sequences, Table S1). The vectors were transfected into 293T cells through the use of the polyetherimide, together with either pCDH or pCDH-FoxM1, as well as phRL-SV40 vector as an internal control. Cells were collected at 36 h after transfection, and both luciferase activities were assessed with the Dual Luciferase Reporter Gene Assay Kit according to the manufacturer’s instructions (Beyotime Biotechnology, China).

### Statistical analysis

All experiments included at least three biological replicates. Comparisons between two groups were analyzed using two-tailed Student’s *t* test. Differences among more than two groups were analyzed using one-way analysis of variance followed by Tukey–Kramer post hoc tests. Values of *p* < 0.05 were considered statistically significant. All data are means ± SD.

### Accession numbers

All sequencing data are deposited in the NCBI GEO database under the accession number: 135486.

## Supplementary information

Supplementary Table 1. The sequence of primers.

Supplementary Figure 1. Loss of FoxM1 has no obvious effect on muscle mass of mice at 2 months of age

Supplementary Figure 2. The effect of FoxM1 on proliferation and differentiation of SCs

Supplementary Figure 3. FoxM1 overexpression results in SC exhaustion and muscle atrophy with age

Supplementary Figure 4. FoxM1 promotes cell cycling of SCs by transcriptionally regulating Ccnb1

Supplementary Figure 5. Inhibition of Cdk1/Ccnb1 partially rescues the decreased SC pool in Pax7-FoxM1 mice

Supplementary Figure 6. Cdh1 is upregulated in activated SCs
