# Supplementary material for: A Cdh1–FoxM1–Apc axis controls muscle development and regeneration
Source: Cell Death Dis. 2020 Mar 9;11(3):180. doi: 10.1038/s41419-020-2375-6 (PMC7062904; doi:10.1038/s41419-020-2375-6)
Supplement: Supplementary file 1 — Supplementary Table 1. The sequence of primers. [file 41419_2020_2375_MOESM1_ESM.doc]

**Supplementary Table 1.** The sequence of primers.

| Primers for genotyping | |
| --- | --- |
| FoxM1 floxp-F1 | TGGCTTCCCAGCAGTACAAATC |
| FoxM1 floxp-R1 | TGCTTACAAAAGACACACTTGGACG |
| FoxM1 floxp-R2 | TCTCGCTCAATTCCAAGACCAG |
| Pax7-FoxM1-F1 | ATTAAGGATCGATCCGGCGC |
| Pax7-FoxM1-R1 | TCTGGGGACCCAGCACTTTC |
| CDH1 floxp-F1 | GGTGCAGGTGAGTCAGAGAGTAA |
| CDH1 floxp-R1 | TGGGGAGAATGCTCTGGGATCA |
| Cre-F | AGCGATGGATTTCCGTCTCTGG |
| Cre-R | AGCTTGCATGATCTCCGGTATTGAA |
| Primers for RT-PCR | |
| mFoxM1-F | TTCTCAAAAGACGGAGGCTG |
| mFoxM1-R | CTGGAAATTTGCAAGAGCTGG |
| mMyoD-F | CGCTCCAACTGCTCTGATG |
| mMyoD-R | ACACAGCCGCACTCTTC |
| mMyf5-F | AGAGGAAGTCCACTACCAT |
| mMyf5-R | GTAATAGTTCTCCACCTGTTC |
| mMyogenin-F | GGTGGAGGATATGTCTGTTG |
| mMyogenin-R | GTGTTAGCCTTATGTGAATGG |
| mMyh3-F | GCTGTTCTTGTGGATGGT |
| mMyh3-R | CTCGTTGGTGAAGTTGATG |
| mAPC-F | TGGAGTATGAAGCAAGGCAG |
| mAPC-R | CCTGGGACTGTAAAAGCTGG |
| mAxin1-F | AGTGATGCTGACACGCTATC |
| mAxin1-R | GGTAAGTGCGAGGAATGTGAG |
| mGSK3b-F | CACCTGCACTCTTCAACTTTAC |
| mGSK3b-R | CACGGTCTCCAGCATTAGTATC |
| mSkp1a-F | CCATGCCTACGATAAAGTTGC |
| mSkp1a-R | CATTTGGTAAAGGAACAGGATCAT |
| mCyclin B1-F | CTGACCCAAACCTCTGTAGTG |
| mCyclin B1-R | CCTGTATTAGCCAGTCAATGAGG |
| mCyclin B2-F | CCTCAGAACACCAAAGTACCAG |
| mCyclin B2-R | CCTTCATGGAGACATCCTCAG |
| mCyclin D3-F | GCGTGCAAAAGGAGATCAAG |
| mCyclin D3-R | GATCCAGGTAGTTCATAGCCAG |
| mCyclin G2-F | GAACAGAGATACCAACCTCGG |
| mCyclin G2-R | GTTTCAGTGCCAGATCCAAAG |
| mCDH1-F | AGAGAAGCCATTGCCAAGTAC |
| mCDH1-R | AACGAATCCCTCAAAGACCG |
| mβ-Actin-F | ACCTTCTACAATGAGCTGCG |
| mβ-Actin-R | CTGGATGGCTACGTACATGG |
| Primers for ChIP-PCR | |
| mCyclin B1-S1 region-F | TACTGAGGCTTCCACTCC |
| mCyclin B1-S1 region-R | TCTCTGTCTCTATTCACTTCTC |
| mCyclin B1-S2 region-F | GGAAGGTGAGAAGTGAATAGA |
| mCyclin B1-S2 region-R | CTCTGACTCTGCCCTCTT |
| mCyclin B1-S3 region-F | CTACCTGGCTGTTGCTAG |
| mCyclin B1-S3 region-R | AGTAACACTAACGCGGATT |
| mCyclin B1-S4 region-F | GCCTTAATAAGGAGTTTGAC |
| mCyclin B1-S4 region-R | AGATTAACATACCAAGCTCC |
| mCyclin B1-S5 region-F | CAGAGGCAGGTGGATTTC |
| mCyclin B1-S5 region-R | TGAGGTTGATAGCATGAAGT |
| mCyclin B1-S6 region-F | AGACCGTCTCTGCAACAA |
| mCyclin B1-S6 region-R | GGGAGTACGCATATCAGTTT |
| mAPC-S1 region-F | AGGTGCATCTGAGGACAG |
| mAPC-S1 region-R | CTCTCCATATCGCTGTAACC |
| mAPC-S2 region-F | GCGATATGGAGAGGACAC |
| mAPC-S2 region-R | TTAATTGCCACTTCAGACAG |
| mAPC-S3 region-F | TAGAAGCGGGATCAGTTTG |
| mAPC-S3 region-R | CGGTGGAGAAGACAGAAAT |
| mAPC-S4 region-F | AAGGTGAGCAGGTTAGAGA |
| mAPC-S4 region-R | GGAATGAGGGATTGGACTG |
| Primers used for subclone | |
| mFoxM1 shRNA-1-F | CCGGCGCTACTTGACATTGGACCAACTCGAGTTGGTCCAATGTCAAGTAGCGTTTTTG |
| mFoxM1 shRNA-1-R | AATTCAAAAACGCTACTTGACATTGGACCAACTCGAGTTGGTCCAATGTCAAGTAGCG |
| mFoxM1 shRNA-2-F | CCGGACTTCCTATTCAGTCCATTAACTCGAGTTAATGGACTGAATAGGAAGTTTTTTG |
| mFoxM1 shRNA-2-R | AATTCAAAAAACTTCCTATTCAGTCCATTAACTCGAGTTAATGGACTGAATAGGAAGT |
| mCDC20 shRNA-1-F | CCGGGCAGCAGAAACGACTTCGAAACTCGAGTTTCGAAGTCGTTTCTGCTGCTTTTTG |
| mCDC20 shRNA-1-R | AATTCAAAAAGCAGCAGAAACGACTTCGAAACTCGAGTTTCGAAGTCGTTTCTGCTGC |
| mCDC20 shRNA-2-F | CCGGCGGAATGACTACTACCTGAATCTCGAGATTCAGGTAGTAGTCATTCCGTTTTTG |
| mCDC20 shRNA-2-R | AATTCAAAAACGGAATGACTACTACCTGAATCTCGAGATTCAGGTAGTAGTCATTCCG |
| mCDH1 shRNA-1-F | CCGGCGTGAACTTCCACAGGATCAACTCGAGTTGATCCTGTGGAAGTTCACGTTTTTG |
| mCDH1 shRNA-1-R | AATTCAAAAACGTGAACTTCCACAGGATCAACTCGAGTTGATCCTGTGGAAGTTCACG |
| mCDH1 shRNA-2-F | CCGGGTGTGGAACCACTCTAGTCTACTCGAGTAGACTAGAGTGGTTCCACACTTTTTG |
| mCDH1 shRNA-2-R | AATTCAAAAAGTGTGGAACCACTCTAGTCTACTCGAGTAGACTAGAGTGGTTCCACAC |
| mFoxM1-cds-5B | CTAGCTAGCATGGACTACAAAGACGATGACGACAAG |
| mFoxM1-cds-3X | CCGGAATTCCTAAGGGATGAACTGAGACCAGTT |
| mCyclin B1-Promoter-F | CGAGCTCGGTCTACCATAGAACAGCTTACT |
| mCyclin B1-Promoter-R | CCGCTCGAGCGATTCGAGAAGACACCCTAACCT |
| mCyclin B1- mutant Promoter-F | ATCCTGGTCAATCGCTAAATCCGCGTTAGTGTTACT |
| mCyclin B1- mutant Promoter-R | AACGCGGATTTAGCGATTGACCAGGATCTCTTGTA |
| mAPC-Promoter-F | CGAGCTCTGCATCTGAGGACAGGCTGGGGAA |
| mAPC-Promoter-R | CCGCTCGAGAGCGCAGGAAGGCGTACATAGGG |
| mAPC-mutant Promoter-F | TCTCTTAAAAATGAACGCAAAATTGAAGGGCCAATTACA |
| mAPC-mutant Promoter-R | CCTTCAATTTTGCGTTCATTTTTAAGAGAGGGCCT |
| GAPDH ChIP-F | CAAGGAGCCAAGACTAGATT |
| GAPDH ChIP-R | TCAAGAGCCTATTGCTAAGT |
