# Supplementary figures and images for: A Cdh1–FoxM1–Apc axis controls muscle development and regeneration
Source: Cell Death Dis. 2020 Mar 9;11(3):180. doi: 10.1038/s41419-020-2375-6 (PMC7062904; doi:10.1038/s41419-020-2375-6)

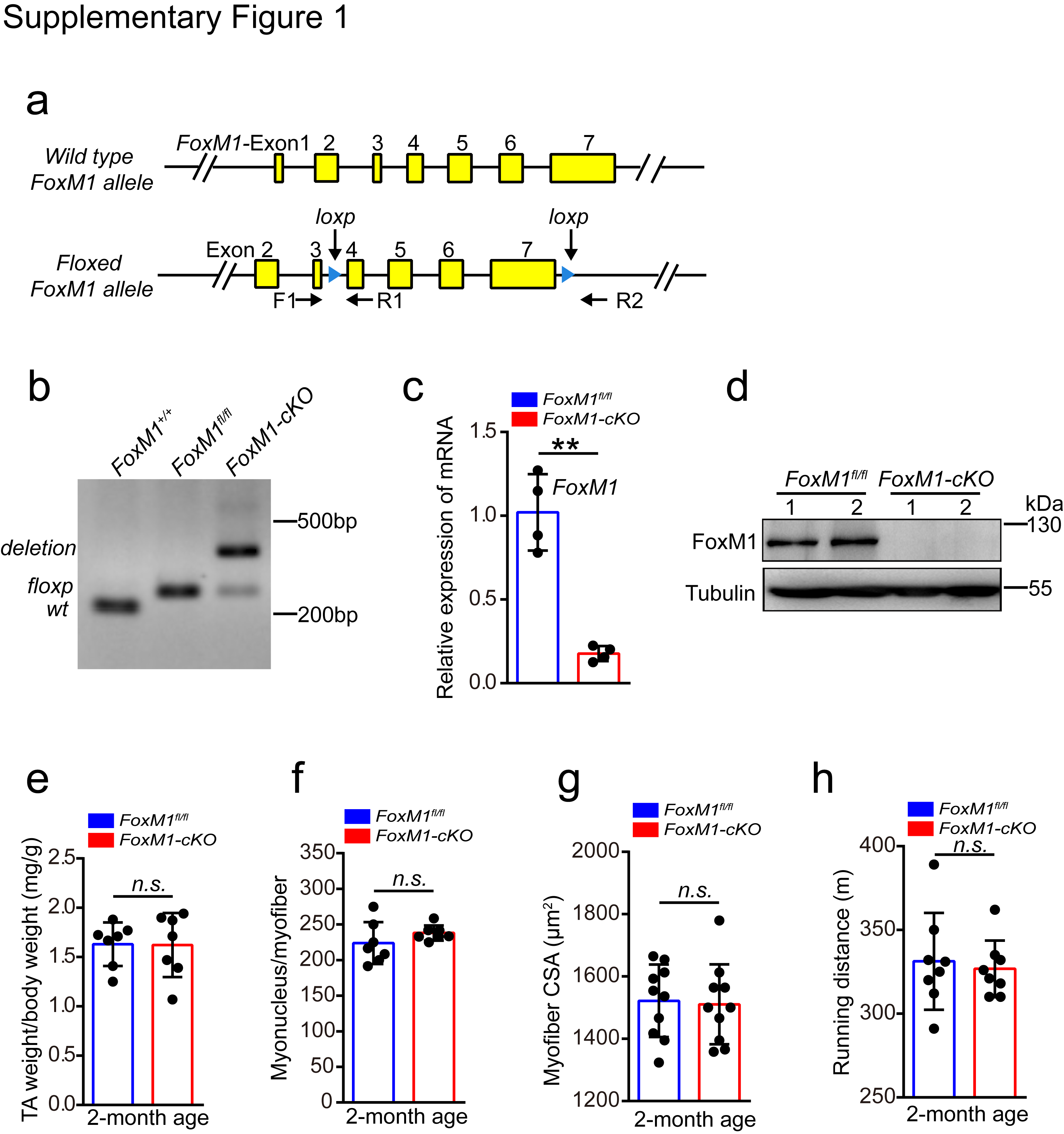

Supplement: Supplementary file 2 — Supplementary Figure 1. Loss of FoxM1 has no obvious effect on muscle mass of mice at 2 months of age [file 41419_2020_2375_MOESM2_ESM.tif]

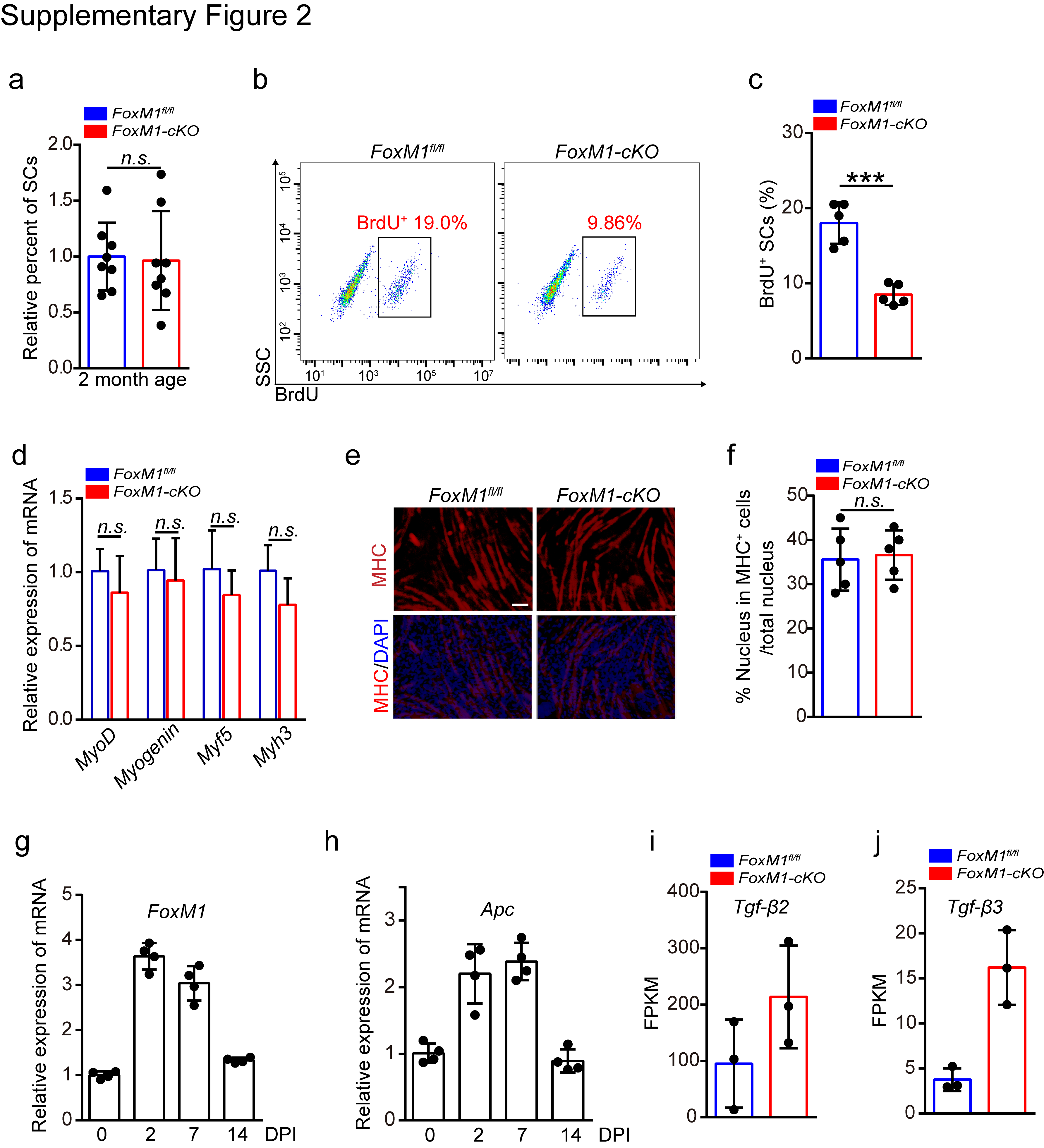

Supplement: Supplementary file 3 — Supplementary Figure 2. The effect of FoxM1 on proliferation and differentiation of SCs [file 41419_2020_2375_MOESM3_ESM.tif]

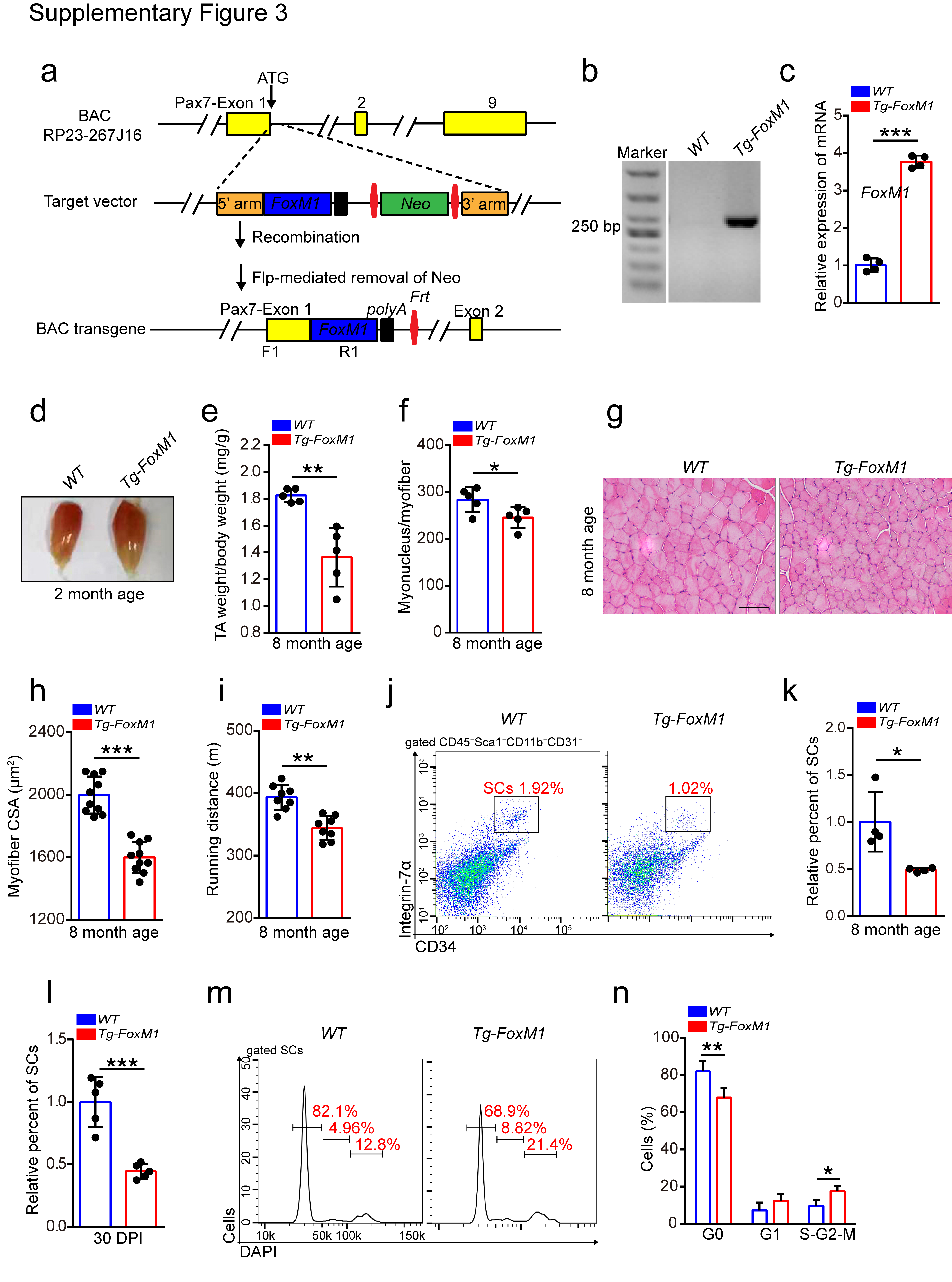

Supplement: Supplementary file 4 — Supplementary Figure 3. FoxM1 overexpression results in SC exhaustion and muscle atrophy with age [file 41419_2020_2375_MOESM4_ESM.tif]

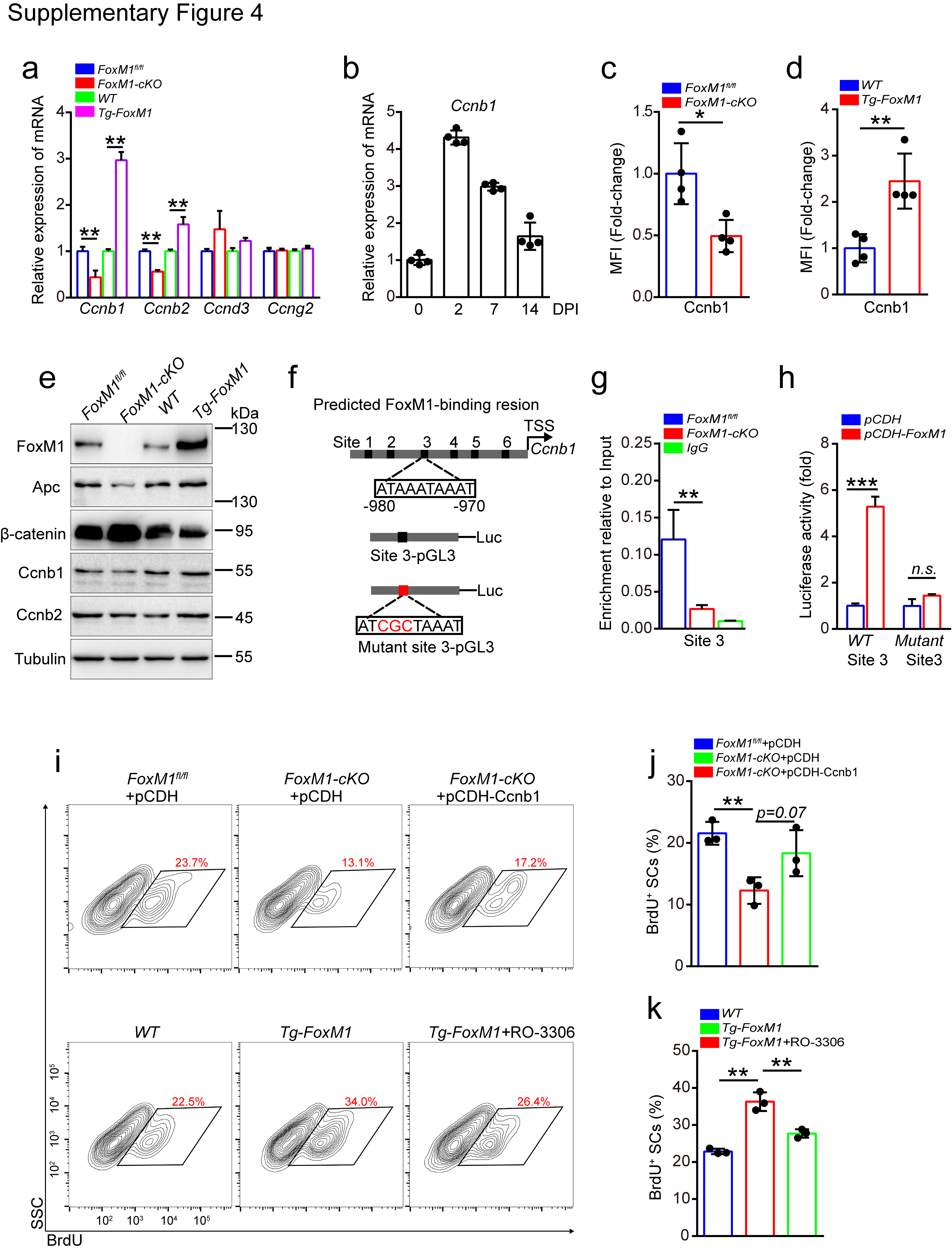

Supplement: Supplementary file 5 — Supplementary Figure 4. FoxM1 promotes cell cycling of SCs by transcriptionally regulating Ccnb1 [file 41419_2020_2375_MOESM5_ESM.tif]

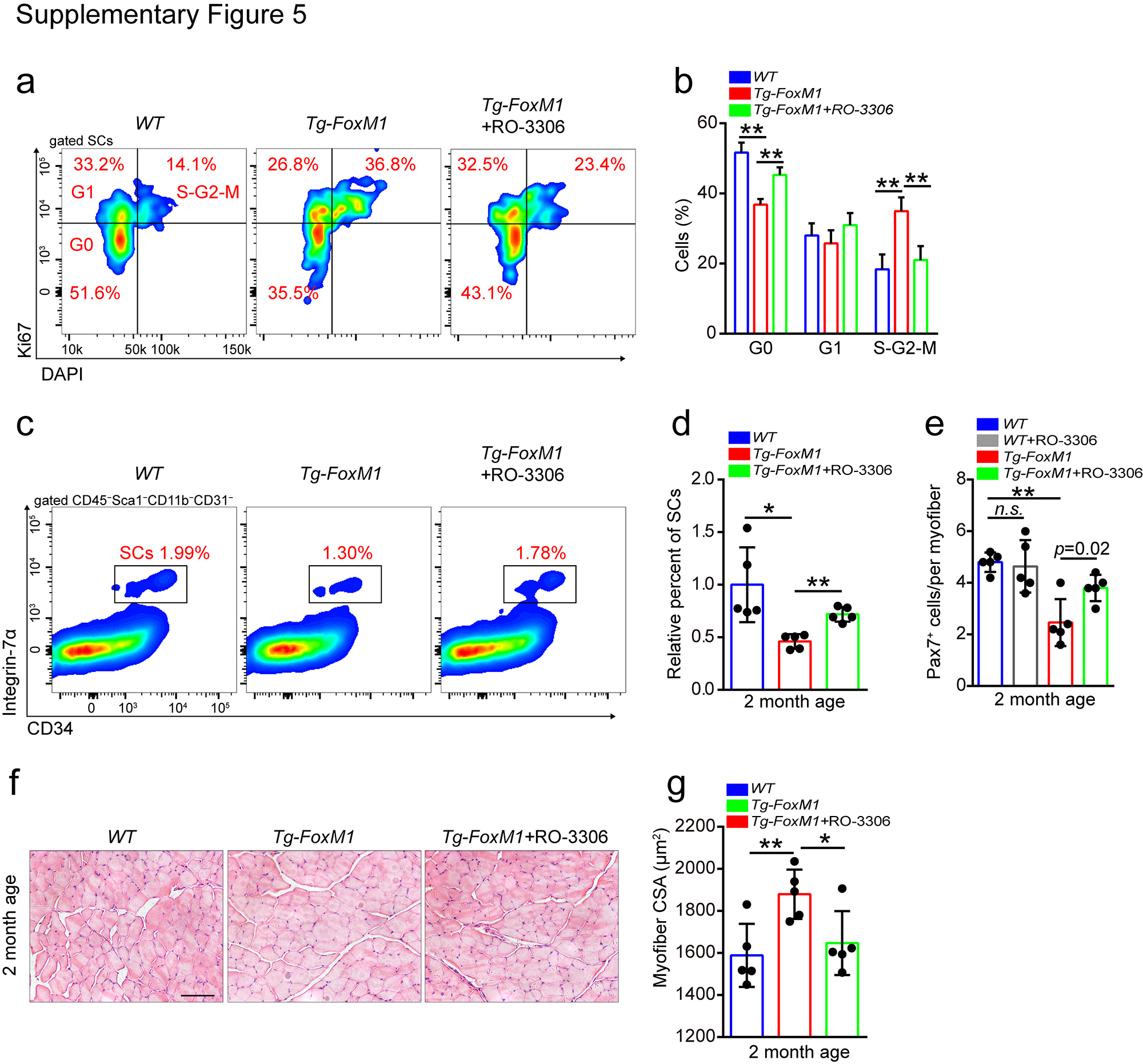

Supplement: Supplementary file 6 — Supplementary Figure 5. Inhibition of Cdk1/Ccnb1 partially rescues the decreased SC pool in Pax7-FoxM1 mice [file 41419_2020_2375_MOESM6_ESM.tif]

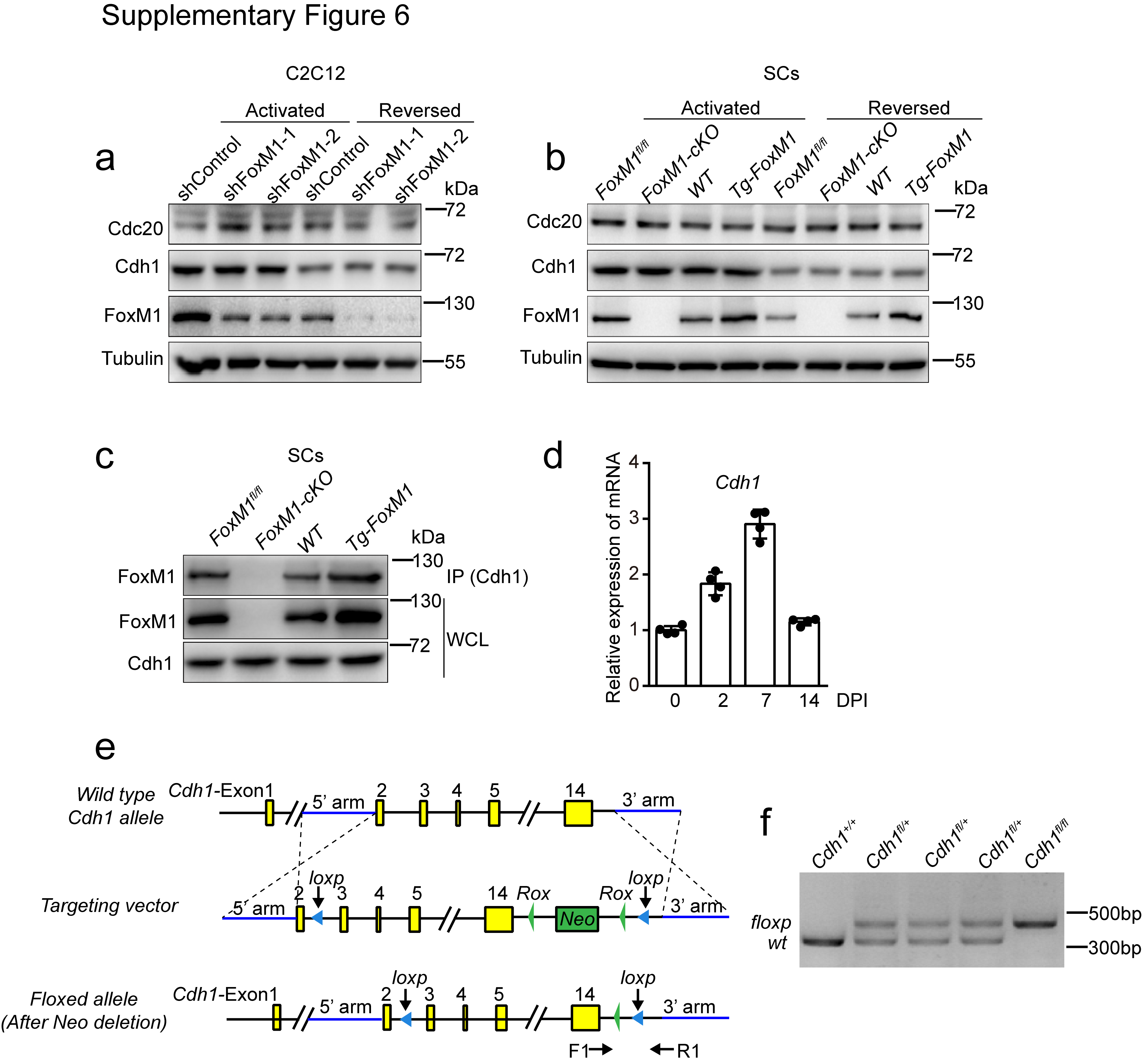

Supplement: Supplementary file 7 — Supplementary Figure 6. Cdh1 is upregulated in activated SCs [file 41419_2020_2375_MOESM7_ESM.tif]
